# Pharmacological and genetic inhibition of ARG2 in CXCR2^Hi^ myeloid-derived suppressor cells combats sepsis-induced lymphopenia

**DOI:** 10.7150/thno.112339

**Published:** 2025-07-11

**Authors:** Qiuyue Long, Shixu Song, Jiwei Li, Jialing Gan, Shuoqi Yang, Boyu Li, Hongli Ye, Binghan Zheng, Fangfang Wu, Zhichen Yu, Jing Wu, Linyu Ding, Mingzheng Jiang, Xiaoyi Hu, Zhancheng Gao, Yali Zheng

**Affiliations:** 1 Department of Respiratory, Critical Care and Sleep Medicine, Xiang'an Hospital of Xiamen University, School of Medicine, Xiamen University, Xiamen 361101, China.; 2 Institute of Chest and Lung Diseases, Xiamen University, Xiamen 361101, China.; 3 Department of Thoracic Surgery, Xiang'an Hospital of Xiamen University, School of Medicine, Xiamen University, Xiamen 361101, China.; 4 Department of Respiratory and Critical Care Medicine, Peking University People's Hospital, Beijing 100044, China.; 5 Department of Cardiology, Xiang'an Hospital of Xiamen University, School of Medicine, Xiamen University, Xiamen 361101, China.; 6 School of Engineering, Westlake University, Hangzhou, Zhejiang 310030, China.; 7 Department of Thoracic Surgery and Oncology, the First Affiliated Hospital of Guangzhou Medical University, State Key Laboratory of Respiratory Disease & National Clinical Research Center for Respiratory Disease, Guangzhou 510120, China.

**Keywords:** single-cell RNA sequencing, septic lymphopenia, myeloid-derived suppressor cell, T cell, arginase 2

## Abstract

**Rationale:** Myeloid-derived suppressor cells (MDSCs) play a critical role in inducing T-cell lymphopenia in sepsis, and the highly heterogeneous MDSCs necessitate the identification of key molecules within these cells.

**Methods:** By integrating bulk and single-cell transcriptomic sequences, we identified the critical molecular and MDSC subpopulation in pneumonia-induced sepsis (PIS) models. Through fluorescence-activated cell sorting (FACS) technology, we isolated the primary target subset to evaluate its immunosuppressive potential via T-cell proliferation assays, and investigate the underlying cellular and molecular mechanisms. To assess the immunological consequences of molecular interventions (pharmacologic blockade and shRNA-mediated knockdown), we employed a “two-hit” experimental model to monitor T-cell-aassociated immune responses and hosts' outcomes following secondary infection. Futhermore, we collected and analyzed clinical samples to support of translating the cellular and molecular concept to human context.

**Results:** We confirmed the specific enrichment of arginase-2 (ARG2) in CXCR2^Hi^ MDSCs, which expanded during sepsis and drove immunosuppression via ARG2-mediated arginine depletion. The blockade of ARG2 and arginine supplements improved the proliferation and decreased apoptosis of CD4^+^ T cells. In PIS models, both ARG2 inhibition and knockdown regained CD4^+^ T cells in lung and bone marrow sites, thus enhancing host's resistance to secondary infections caused by opportunistic pathogens. Further mechanistic investigations indicated p38-MAPK as a critical regulator of the protein stability of the immunosuppressive molecule ARG2 in CXCR2^Hi^ MDSCs, particularly in response to lipopolysaccharide (LPS) stimulation. In the human context, we revealed that CXCR2^Hi^ MDSC increased in peripheral in septic patients and correlated significantly to lymphopenia and elevated ARG2 levels.

**Conclusions:** Sepsis stimulated p38-MAPK signaling and expanded ARG2-enriched CXCR2^Hi^ MDSCs to mediate septic lymphopenia via arginine depletion. The ARG2 inhibition restored T-cell immunity against secondary infection in septic immunosuppressed hosts. These findings identified CXCR2^Hi^ MDSC-derived ARG2 as a promising target of immune enhancement therapy in sepsis.

## Introduction

Sepsis is a syndrome of life-threatening organ dysfunction caused by the host's systemic inflammatory and dysregulated immune responses to infection 1. Despite the standard implementation of timely antibiotics and fluid resuscitation since the 1990s 2, the mortality rate of septic patients remains as high as 20-30%, accounting for 20% of all global deaths annually 3. The failures of therapeutic strategies that target excessive inflammation in clinical trials, coupled with the deepening understanding of the immune system's central role in septic pathology 4, underscores the urgent need for novel immunotherapeutic approaches. These innovative treatments hold the potential to significantly reduce the persistently high mortality rates associated with sepsis worldwide. The World Health Organization's designation of sepsis as a global health priority in 2017 5 further emphasizes the imperative for new solutions that address the complex immunological challenges posed by this condition.

Sepsis is marked by a profound disruption of both innate and adaptive immunity, often leading to a transition from an initial hyperinflammatory state to a subsequent immunosuppressive state, characterized by persistent lymphopenia—a condition associated with increased susceptibility to secondary infections and higher mortality rates in septic patients 6-8. Despite the recognition of lymphocyte apoptosis, particularly of CD4^+^ T cells, as a key process in septic lymphopenia, the intricate mechanisms driving this phenomenon are not fully elucidated. Studies have demonstrated lymphocyte apoptosis in both cellular and animal models induced by gram-negative bacteria-derived LPS 9, as well as in septic patients 10, 11. Early strategies aimed at mitigating septic progression by inhibiting apoptotic signaling pathways were thought to be promising 12. However, the multifaceted physiological roles of caspases, which are central to apoptosis, have complicated the development of targeted therapies, as their inhibition could interfere with vital cellular processes 13, 14. Consequently, further research is needed to explore the cellular and molecular mechanisms underlying septic lymphopenia to uncover potential therapeutic targets.

In the context of infectious diseases, including *mycobacterium tuberculosis*, *klebsiella pneumoniae* (*K.p*), and *staphylococcus aureus* 15-17, the emergence of MDSCs from immature myeloid cells during emergency granulopoiesis has been linked to lymphopenia and immunoparalysis. MDSCs are known to produce a variety of immunosuppressive mediators, such as arginase-1 (ARG1) and nitric oxide, which deplete L-arginine, an essential metabolite for T-cell proliferation 18. Additionally, MDSCs can express programmed death 1 ligand (PD-L1), contributing to T-cell exhaustion and dysfunction through interactions with programmed cell death protein 1 (PD-1) on T cells 19. Clinical studies have consistently shown an expansion of MDSCs following sepsis, with a correlation between increased MDSC proportions or numbers and disease severities, as well as the incidence of secondary infections. The emergence of MDSCs is shaped by diverse factors, including pathogen types, infection sites, and the host's immune state 20, leading to a heterogeneous population with diverse immunophenotypes and molecular functions. In a clinical study, both monocyte-derived MDSCs (M-MDSCs) and granulocyte-derived MDSCs (G-MDSCs) accumulated in septic patients, but only the increase in G-MDSCs was associated with secondary infections and sepsis reoccurrence 21. This suggests that the immunosuppressive mechanisms of MDSCs are not uniformly activated; instead, their impact is determined by the predominant MDSC subtype, the pathological context, and the specific immunosuppressive site. Thus, our study aimed to characterize and investigate the functional subtypes of MDSCs during the progression of PIS, which can be recognized as potential therapeutic targets.

In this study, we identified ARG2-enriched CXCR2^Hi^ MDSCs as a pivotal immunosuppressive subset in a *K.p*-induced PIS model. Following PIS, this subset accumulated in the lung and bone marrow (BM), mediated arginine starvation in an ARG2-dependent manner, further suppressing CD4^+^ T cell proliferation and survival. In septic patients, this subset significantly expanded in the peripheral blood, exhibiting significant positive correlations with neutrophils and negative correlations with lymphocytes. Utilizing a “two-hit” mouse model, we demonstrated that pharmacological blockade and *in vivo* knockdown of ARG2 enhanced T cell immune responses against secondary infections by restoring CD4^+^ T cells, reducing bacterial loads and improving survival rates. Our findings underscore the role of ARG2-enriched CXCR2^Hi^ MDSCs in the pathogenesis of septic lymphopenia and propose ARG2 as a promising therapeutic target for immunomodulation in sepsis.

## Methods

### Bacteria strains

The highly virulent *K.p* strain ATCC 43816 was purchased from the American Type Culture Collection. The clinical strain F132, a sequence type 11 carbapenemase-producing *Klebsiella pneumoniae* (CP-Kp) strain with extensively drug-resistant phenotype 22, was isolated from an ICU-ward inpatient with nosocomial infection and used to induce secondary opportunistic infections in the two-hit model. Both strains were cultured in a lysogeny broth medium in the biosafety level 2 laboratory.

### Animal models

Specific pathogen-free (SPF) c57BL/6 mice aged 7-9 weeks were purchased from the Xiamen University Laboratory Animal Center, Xiamen, China. All experiments used female c57BL/6 mice to maintain relatively hormonal consistency when studying lymphopenia dynamics. The mice were healthy and were housed in individually ventilated cages for one week's habituation before experiments, with standard feeding conditions of temperature (23 ± 3 °C), relative humidity (55 ± 5%), and a 12-h light/dark cycle. The animal project has been reviewed and approved by the Ethics Committee of Laboratory Animals of Xiamen University (Approval number: XMULAC20210021).

The lethal pneumonia-associated sepsis mice model was established according to the previous study 23. Briefly, after anesthetization with tribromoethanol (250 mg/kg), mice were intratracheally inoculated with *K. p* strains ATCC43816 (about 1 × 10^4^ colony-forming units) to induce the pneumonia onset and the progression to sepsis. At given time points, the anticoagulated peripheral blood (PB) and BM cells were harvested, which were then slowly spread onto the 1.084 g/mL Ficoll medium (GE, 17-5446-02) and centrifuged at 350 × g for 30 min at 20 °C to obtain low-density PB and BM cells. This was followed by erythrocyte lysing with ACK lysis buffer (Sbjbio, BL-O51) at room temperature (RT) and cell washing with pre-chilled PBS buffer, adding 2% fetal bovine serum (FBS). The viability of retained cells was assessed using trypan blue under microscopy. After assuring that the cell viability was more than 88%, isolated PB and BM samples were stored in liquid nitrogen for single-cell sequencing. To approximate the clinical scenario of human sepsis, where secondary infections often occur following a period of immunosuppression, mice were treated with the antibiotic levofloxacin (Selleck, S1940, 15 mg/kg) at 48 and 72 h post-infection. This treatment allowed the mice to survive until Day 6, when they were subjected to a secondary infection with the clinical strain F132 at the inoculum dose of 1×10⁷ CFU, the highest tolerated dose for healthy mice (Figure S6J).

### Adeno-associated virus (AAV) delivery

A recombinant adeno-associated virus (AAV2/8) for the knockdown of ARG2 in CD11b^+^ cells was purchased from OBiO Technology (Shanghai, China). Each mouse was treated with 50 μL of 3 × 10^11^ viral genome copies AAVs via the tail vein, in which AAV2/8-CMV-mCherry-FLAG-WPRE was CMV-mCherry group and AAV2/8-CD11b promoter-mCherry-mir30shRNA-WPRE was CD11b-shArg2-mCherry group. After 4 weeks of AAVs injection, BM cells were collected for the verification of ARG2 knockdown and the subsequent* in vivo* experiments were initiated.

### Human samples

The ethics approval for human samples was authorized by the ethics committee of the School of Medicine, Xiamen University (Approval number: XDYX202302K06). All subjects or their guardians provided informed consent before sample collection. The peripheral blood samples were collected from age- and gender-matched healthy volunteers (n = 5), pneumonia patients (n = 12), and septic patients (n = 10) in Xiang'an Hospital of Xiamen University from October 10, 2023 to April 20, 2024 (Table S6). According to the Sepsis 3.0 definition, sepsis was confirmed by infectious diagnosis and sequential organ failure assessment (SOFA) score of two points or more 1. For pneumonia, patients were diagnosed by criteria of community-acquired pneumonia in terms of symptoms, physical signs, chest radiography, and laboratory tests 24. The exclusion criteria included those under 18 years, pregnant, hematological malignancies, and those who received immunosuppressive therapy in the past 15 days. A total of 2-4 mL of EDTA-anticoagulated blood was collected within 72 h of hospitalization and treated within 24 h. After centrifugation at 1, 500 rpm for 10 min, the plasma and leukocyte were retained and frozen in liquid nitrogen until use.

### Pathological evaluation

Lung, liver, and kidney tissues were harvested and fixed in 4% paraformaldehyde (PFA, Greagent, 30525-89-4) for 72 h at RT for pathological evaluation. For hematoxylin and eosin (H&E) staining, fixed tissues were dehydrated in graded alcohol and embedded in paraffin wax followed by preparation of 5 µm thickness of tissue slices, dewaxing, and H&E staining. At last, the tissue histopathology was captured by an automatically scanning microscope Motic VM1 (Motic, China) with magnifications from 20 to 200 times.

For micro-CT imaging, the 4% PFA was first injected endotracheally to inflate the lung. Then, the entire cardiopulmonary tissue was collected and fixed in 4% PFA for 24 h, and immersed in 97% ethanol for 72 h. When imaging, the samples were dried on gauze at room temperature. Setting X-ray parameters of 80 kV voltage and 12 µm adequate pixel size, micro-CT images were acquired by SKYSCAN 1272 (Bruker, Belgium). Next, CT images of individual samples were reconstructed using the NRecon software. The final images were displayed and exported by the Data Reviewer software.

### Bulk-RNA sequencing and analysis

Total RNA was extracted from 1×10^6^ cells using the TRIzol reagent. The sample was quantified using the NanoDrop spectrophotometer (Thermo Fisher Scientific, USA), and RNA integrity was determined using the Agilent 2100 Bioanalyzer (Agilent Technologies, USA). The samples with an RNA integrity number (RIN) value ≥ 7 were kept to library construction and sequencing with the PE100 strategy (BGISEQ, China). Differentially expressed genes (DEGs) were identified from the gene expression matrix by the “DESeq2” package, with criteria of |log2FC| > 1 and false discovery rates (FDR) < 0.05. DEGs were conducted for the enrichment analysis through the “clusterProfiler” package based on the GO database. Then enriched items were eliminated redundancy and visualized via Revigo 25. The RNA sequencing data was deposited in the China National Center for Bioinformation platform by access number CRA015979.

### Single-cell RNA sequencing, pre-processing, and clustering

In brief, individual cells were determined and captured by 10× Genomics-based droplet sequencing technology. Library construction was performed with the single cell 3' library kit (10× Genomics, 1000268) following the manufacturer's instruction and sequenced with paired-end reads through the MGISEQ-2000RS (BGISEQ, China). Then the reads were mapped to the mouse genome mm10 with default parameters.

Based on the strict quality control procedure, low-quality cells in single-cell datasets were identified and filtered out as follows: (1) cells with less than 200 genes or greater than 90% of the maximum quantity of detectable genes, (2) cells with more than 15% proportion of mitochondrial unique molecular identifiers (UMI). Following the removal of these low-quality cells, PB and BM Seurat objects contained 26,088 and 30,171 cells, respectively, for subsequent analysis. According to the standard pre-processing workflow by “Seurat” package 26, filtered UMI counts were firstly normalized by “ScaleData” function with a scaling factor of 10,000. Next, the 2,000 highly variable genes generated by the “FindVariableFeatures” function were subjected to principal component analysis (PCA) by the “vst” method of “FindNeighbors” function. The “ElbowPlot” function was used to determine the appropriate number of principal components for cell clustering. The subpopulations were then yielded from cell clustering by the uniform manifold approximation and projection (UMAP) method of “FindClusters” function when setting resolution = 0.8, which were further annotated identities in manual with typical cell-type markers and displayed in two-dimension space. With the “FindMarkers” function, DEGs among subpopulations and disease groups were obtained when setting log2FC > 0.25, minPct > 0.1, and Padj < 0.05. The single-cell RNA sequencing data was available on the GEO platform by access number GSE262512.

### Cell communication analysis

To infer possible cell-cell communication molecules, we conducted cell interaction analysis based on the “Cellchat” 27, a recognized repository that included signaling molecules and preset algorithms involving receptor-ligand interactions. The communication probability was estimated based on the average expression of ligand-receptor pairs in the normalized single-cell matrix. A *p* < 0.05 in the permutation test was considered interaction significant.

### External database analysis

To investigate the clinical relevance of the ARG2 enriched MDSC subset, we further analyzed public bulk- and sc-RNA sequencing datasets from clinical patients, which were downloaded from GEO (GSE186054, GSE136200, GSE5273, GSE28750, GSE655682, GSE57065, GSE54154, GSE63042) and ArrayExpress (E-MTAB-4421) databases (Table S3). Before analysis, the gene expression matrix in each dataset was log-transformed and normalized. The relative log expression (RLE) was used for quality control 28. The receiver operating characteristic (ROC) curves were obtained with the “pROC” package, while area under curve (AUC) values were applied to assess the diagnostic value of the gene set representing the target subset. The odds ratio (OR) for 28-day mortality was calculated via the Logistic univariate regression in “survival” and “survminer” packages. The |OR| > 1 and *p* < 0.05 were considered as judgement criteria of risk factors.

### Flow cytometric sorting and analysis

BM cells were sterilely harvested from femurs and tibias of c57BL/6 mice and processed with a 70 µm strainer (Biosharp, BS-70-XBS). The erythrocytes were lysed with ACK lysis buffer. The cells were then resuspended at 1 × 10^6^/mL, followed by blocking with anti-mouse FcR reagent (Miltenyi, 130-092-575) at 4 °C in the dark for 15 min. After centrifugation at 300 g for 5 min at 4 °C and washed once with PBS, cells were incubated on ice for 40 min in flow cytometry staining buffer (PBS adding 1% FBS and 1 mM EDTA) with a combination of fluorescently labeled antibodies as follows: APC-CD11b (BioLegend, 101212), PE-Gr1 (Proteintech, PE-65140), and PE/Cyanine7-CXCR2 (CD182) (BioLegend, 149315). The BM cells were sorted by MoFlo Astrios EQS (Beckman Coulter, USA) or analyzed by CytoFlex LX cytometer (Beckman Coulter, USA). For CD4^+^ T cell analysis, cells from bronchoalveolar lavage fluid (BALF), spleen, blood, and BM were stained with fluorescently labeled antibodies to APC-CD3 (BioLegend, 100236), PE-CD4 (BioLegend, 100406), PE/Cyanine7-CD4 (Elabscience, E-AB-F1097H), FITC-Annexin V (Beyotime, C1062S), and DAPI (Sigma-Aldrich, D9542, 4 μg/mL).

For clinical sample analysis, the thawed leukocyte was cultured overnight at 37 °C and 5% CO_2_ in RPMI 1640 medium (Gibco, 11875101) adding 10% FBS (Gibco**,** A5670501). On the second day, cells were collected and adjusted to a concentration of 1 × 10^6^/mL. After blocking nonspecific binding by anti-human FcR (CD16/CD32) antibodies (BioLegend, 422302), cells were surfaced stained with fluorescently labeled antibodies to APCH7-HLA-DR (BD, 561358), BB515-CD33 (BD, 564588), PECy7-CD11b (BD, 557743), and PE-CXCR2 (BioLegend, 320705) in 200 μL of flow cytometry staining buffer on ice for 40 min. Next, the cells were washed with PBS and stained with fixable viability Stain 700 (BD, 564997, AF700) in the dark at room temperature for 10 min. Finally, the cells were washed again and resuspended in a PBS buffer for flow cytometry analysis. For each sample, at least 10, 000 events were recorded and processed via FlowJo software v10.

### T-cell proliferation assays

Activated T-cell proliferation capacities were suppressed by BM-derived MDSCs. T cells were sorted from splenocytes of healthy c57BL/6 mice by the CD3 marker and stained with 2 μM 5(6)-carboxyfluorescein diacetate succinimidyl ester (CFSE, BestBio, BB-4211) in PBS at 37 ℃ for 10 min following the manufacturer's instructions. The RPMI 1640 medium with 10% FBS was added to stop the staining process. After placing on ice for 5 min and washing, CFSE-labelled T cells were seeded in U-bottom 96-well plates at 2 × 10^5^ cells/well in the presence of 5 μg/ml CD3ε (BioLegend, 100339) and 5 μg/ml CD28 (BioLegend, 102115) antibodies for 3 days, with the co-culture of BM-derived MDSCs at the ratio of 1:1 or 2:1. The suppressive efficiency of T-cell proliferation was then determined by flow cytometry based on the fluorescence strength of CFSE. The BEC hydrochloride (MedChemExpress, HY-19548A, 0.625-10 μM) and L-arginine (MedChemExpress, HY-N0455, 100 μM) were utilized to inhibit the suppressive capabilities of MDSCs.

### Cytokine and arginine measurement

Concentrations of arginine (Cloud-Clone Corp, CEB938Ge), arginase-2 (Shanghai YSRIBIO industrial co., LTD., CS-3226E), and cytokines (all purchasing from ABclonal) included IFN-γ (RK00019), IL-4 (RK00036), IL-17A (RK00039), IL-6 (RK00008), IL-10 (RK00016), and CXCL2 (RK04208) were measured through enzyme-linked immunosorbent assay according to instructions of commercial ELISA kits. The concentrations were examined at 450 nm wavelength of absorbance in the Mutiskan SkyHigh microplate reader (Thermo Fisher Scientific, USA) and computed based on standard curves (R^2^ > 0.99).

### Quantitative real-time PCR (RT-PCR)

Total RNA was extracted from 1 × 10^6^ cells with 1 mL TRIzol reagent (Beyotime, R0016). The cDNA was synthesized from 500 ng RNA using Evo M-MLV master mix (Accurate Biotechnology, AG11706) by the reverse transcription procedure. The RT-PCR procedure was achieved by using a thermal cycle meter (BIO RAD CFX96, USA) and the SYBR Green premix reagent (Accurate Biotechnology, AG11701). Based on the 2^-ΔΔCT^ method, mRNA quantities were normalized to housekeeping gene *Gapdh* and finally displayed as relative expression levels. The primer sequences of mouse species used in this study were listed in Table S7.

### Western blot

At least 20 μg proteins were separated in precast gradient gels by SDS-PAGE electrophoresis and transferred onto methanol-activated PVDF membranes (Merck Millipore, ISEQ00010). The PVDF membranes were then incubated with primary antibodies at 4 °C overnight and subsequent with HRP-goat anti-rabbit (Proteintech, RGAR001) or HRP-goat anti-mouse (Proteintech, SA00001-1) secondary antibodies (1:10000) at RT for one hour. After washing three times with TBST buffer, the chemiluminescent substrate was laid on PVDF membranes, and target proteins linked to primary antibodies were visualized with the chemiluminescence imaging system Azure C300 (Azure Biosystems, USA). Primary antibodies applied in this study included Actin (81115-1-RR, 1:5000), ARG2 (14825-1-AP, 1:2000), phospho-p38 (28796-1-AP, 1:1000), and p38 (66234-1-Ig, 1:1000) purchasing from Proteintech, as well as ARG1 (A4923, 1:2000) and PD-L1 (A11273, 1:1000) purchasing from ABclonal.

For cell stimulation experiments, sorted CXCR2^Hi^ MDSCs were seeded in six-well plates (LABSELECT, 11110) at 2 × 10^6^ per well and treated with 1 μg/mL LPS (Sigma-Aldrich, L2880) or 10 ng/mL IFN-γ (ABclonal, RP01070) for 5 h. The JSH-23 (TargetMol, USA, T1930), LY294002 (MedChemExpress, HY-10108), IPI-594 (MedChemExpress, HY-100716), Doramapimod (TargetMol, USA, T6308), Tofacitinib (TargetMol, USA, T6321), MG-132 (TargetMol, USA, T2154), and Cycloheximide (MedChemExpress, HY-12320) were used to pre-treated the cells at a concentration of 10 μM for 1 h before LPS stimulation.

### Statistical analysis

Statistical analyses and visualization of transcriptomic datasets were conducted by R software (Version 4.2.1). Column bar graphs were plotted using GraphPad Prism v9. Statistical tests were selected based on the data distribution and its variability. Unless indicated in the figure legends, results were expressed as mean ± standard deviation (SD). All *in vitro* experiments were performed with at least three independent biological replicates, and all individual data points (including technical replicates from each experiment) were analyzed without aggregation or exclusion. For *in vivo* studies, each group included at least three biologically independent mice, and all observations were retained for statistical analysis. When comparing two groups, the two-sided Student's t-test and the Wilcoxon test were applied for parametric data and nonparametric data, respectively. When comparing more than two groups, the one-way analysis of variance (ANOVA) was conducted for parametric data, and the Kruskal-Wallis test was conducted for nonparametric data. In this work, a *p-value* of less than 0.05 was considered statistically significant (*p < 0.05, **p < 0.01, ***p < 0.001). All experiments were repeated at least in triplicate, and the representative one replicate of three independent experiments.

## Results

### Bulk-RNA sequencing revealed elevated arginase-2 involved in T cell proliferation during pneumonia-induced sepsis

**Figure 1:**
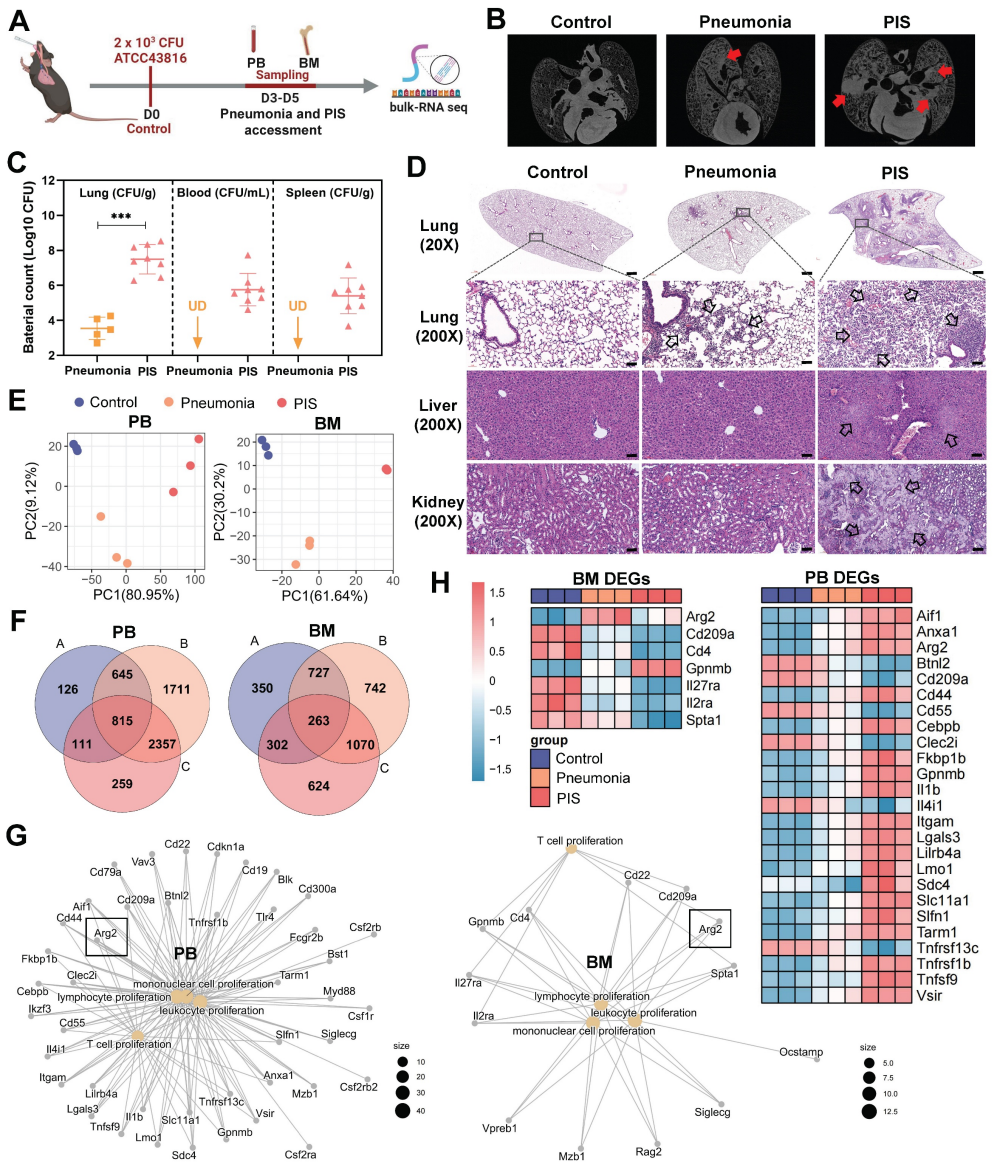
** Bulk-RNA sequencing revealed elevated arginase-2 involved in T cell proliferation during pneumonia-induced sepsis (PIS). (A)** Collection of peripheral blood (PB) and bone marrow (BM) samples from klebsiella pneumoniae induced PIS models for bulk-RNA sequencing. **(B)** Representative micro-CT images of pneumonia in mice cardiopulmonary tissues. Red arrows marked the pneumonic lesion site. n = 5-8 biologically independent mice.** (C)** Bacterial loads of lung, blood, and spleen samples in pneumonia and PIS mice. UD meant undetectable. **(D)** HE staining showed inflammatory infiltration and tissue injury in model groups, 20× (scale bar: 500 μm) and 200× (scale bar: 50 μm), respectively. Black arrows indicated pathological areas.** (E)** PCA plots of bulk-seq data showed transcriptional characteristics of PB and BM samples. **(F)** Venn diagram for differentially expressed genes (DEGs) in PB and BM samples. DEGs were generated from two-by-two comparisons. A: pneumonia vs. control, B: PIS vs. pneumonia, and C: PIS vs. pneumonia. **(G)** The network map of gene-function relationships depicted DEGs corresponding to biological processes associated with leukocyte proliferation in PB and BM. **(H)** Heatmaps showing DEGs enriched in the T cell proliferation process in PB and BM, where *Arg2* was upregulated in pneumonia and PIS conditions. Data were presented as mean ± SD. ***p < 0.001.

To explore the molecular mechanisms contributing to lymphopenia in PIS, we constructed a solid PIS model. Mice were intratracheally inoculated with *K.p*, a common etiology of pneumonia and sepsis, to induce pneumonia. An inoculation dose of 2×10^3^ CFU *K.p* (ATCC43816) was confirmed as a half-death dose (Figure S1, A and B). Post-inoculation, the infected mice were categorized into pneumonia and PIS groups based on the low body temperature (< 32℃) and behaviors items corresponding to the “mouse clinical assessment score for sepsis” (M-CASS) (Table S1 and S2) 29, 30. Micro-computed tomography (micro-CT) imaging revealed pronounced pulmonary infiltrations in the PIS group relative to the pneumonia group (Figure 1B). Consistent with this, elevated levels of pro-inflammatory cytokines (including TNF-α, IL-6, and IL-1β) in BALF, as well as decreased lymphocytes in peripheral blood, were observed in the PIS group (Figure S1, C and D). Furthermore, we also compared bacterial loads across various organs harvested from the PIS and the pneumonia group. As shown in Figure 1C and Figure S1F, compared to the pneumonia, the bacterial load in lungs is significantly elevated in PIS mice (3.80-log higher CFUs, *p* < 0.01), with detectable *K.p* in peripheral blood and distant spleens, indicating a systemic dissemination of infection. Biochemical assays (Figure S1E) and histopathological examination of major organs (Figure 1D) revealed multi-organ dysfunction in the PIS group. Collectively, the above evidence of infection and multi-organ dysfunction, collectively validating the reliability of our PIS model to mimic the complexities of clinical sepsis 1.

Given that BM has been reported to be the primary site of T cell proliferation during recovery from septic lymphopenia 31, we collected low-density immune cells in PB and BM at D3-D5 post-intubation for bulk sequencing (Figure 1A). Principal component analysis demonstrated distinct transcript clusters among control, pneumonia, and PIS groups (Figure 1E). Differentially expressed genes (DEGs) were identified by comparing pneumonia versus control, PIS versus control, and PIS versus pneumonia, respectively. After intersection analysis, a total of 815 and 263 key DEGs were identified in PB and BM, respectively (Figure 1F). Enrichment analysis revealed that these PB and BM DEGs were predominantly involved in leukocyte proliferation regulation, cell killing, and tumor necrosis factor production (Figure S1G). Focusing on the regulation of leukocyte proliferation, we constructed network maps of gene-function relationships depicted in Figure 1G. Among the DEGs involved in T cell proliferation, *arginase-2* (*Arg2*) demonstrated a noticeable upregulation in both PM and BM samples as the disease progressed (Figure 1H). This pattern of increased expression initially implicates a potential role for *Arg2* in modulating T lymphocyte proliferation within the context of sepsis.

### Single cell-RNA sequencing identified high ARG2 expression in CXCR2^Hi^ MDSC

**Figure 2:**
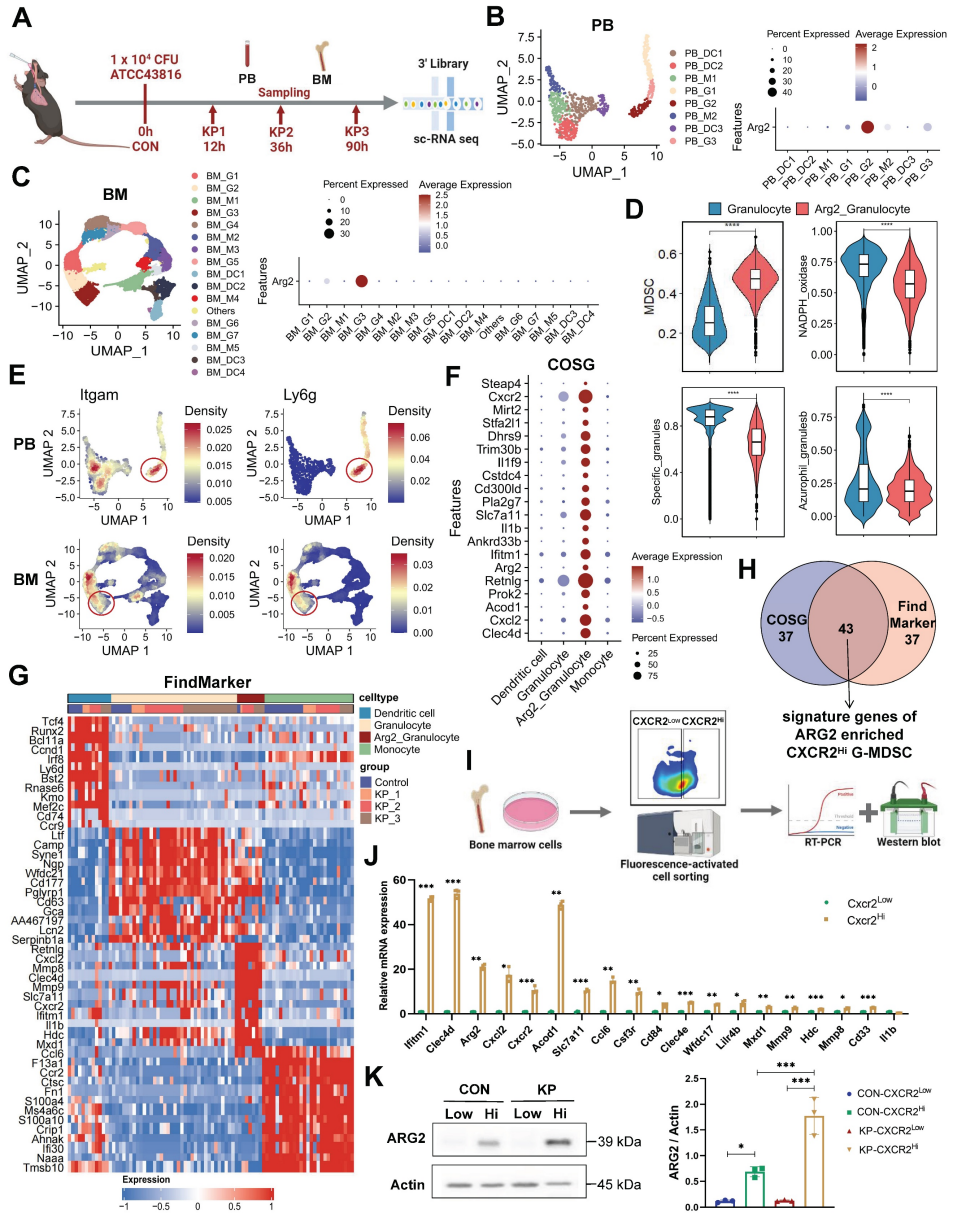
** Single cell-RNA sequencing identified high *Arg2* expression in CXCR2^Hi^ myeloid-derived suppressor cell (MDSC). (A)** Flowchart of the sample collection from PIS animal at the healthy (CON), local pneumonia (KP1), distant spread (KP2), and ultimate sepsis status (KP3) for sc-RNA sequencing. **(B)** The two-dimensional UMAP distribution for 881 myeloid cells in PB, with the dominant *Arg2* expression in the PB_G2 cluster. **(C)** The two-dimensional UMAP distribution for 22, 894 myeloid cells in BM, with the dominant *Arg2* expression in the BM_G3 cluster. **(D)** Compared functional scores of *Arg2* enriched granulocytes and other granulocytes. **(E)** Feature plots indicated that *Arg2-enriched* granulocytes (red circles) belonged to MDSCs, as marked by *Itgam* (*Cd11b*) and *Ly6g* (*Gr1*). **(F)** The top 20 genes specially expressed in ARG2 enriched MDSCs (combing PB_G2 and BM_G3) by COSG analysis, including cell surface marker *Cxcr2*. **(G)** The heatmap exhibited genes highly expressed in ARG2-enriched MDSCs by FindMarker analysis, with a high abundance of *Cxcr2* transcripts. **(H)** Venn diagram showing the generation of signature genes of the ARG2-enriched MDSCs. **(I)** Overview of sorting approach of CXCR2^Low^ and CXCR2^Hi^ subsets in BM-derived MDSCs. **(J)** RT-PCR assay validating the high expression of signature genes in the CXCR2^Hi^ subset from CON mice. **(K)** The protein level of ARG2 by western blotting and quantitative bar charts. Data were represented as mean ± SD. Statistical significances were analyzed using an unpaired t-test. *p < 0.05, **p < 0.01, ***p < 0.001.

To determine the cellular origin of *Arg2*, we conducted single-cell transcriptome sequencing (sc-RNA seq) on PB and BM cells from PIS mice. A lethal dose of 10^4^ CFU *K.p* ATCC43816 was intratracheal inoculated to induce PIS 29, 32. Using the body temperature and M-CASS scoring systems, we categorized mice into stages of local pneumonia (KP1), systemic spread (KP2), and sepsis (KP3), which correspond to approximately 12 hours, 36 hours, and 90 hours post-inoculation, respectively (Figure S2A). Serial samples of PB and BM from PIS mice were harvested for sc-RNA seq analysis, as outlined in Figure 2A. After quality control, a total of 26,088 PBMCs and 30,171 BMMCs were retained for downstream analysis. Unsupervised clustering and cell type identification analysis were performed using Seurat 26. In PB cells, the uniform manifold approximation and projection (UMAP) yielded five unique immune cell types, including the CD8 T, CD4 T, B, natural killer (NK), and myeloid cells (Figure S2, B and C). In BMMCs, the identified cell types included myeloid, hematopoietic, T, B, and natural killer cells (Figure S2, D and E). Our analysis revealed a dynamic and complex interplay among various immune cell populations in response to sepsis. Early in the disease course, there was a notable decrease in myeloid cells within the BM, which corresponded with a peak in their presence in the PB as the mice progress to a septic state (Figure S2, F and G). Similarly, B and NK cells exhibited a trend of decreased frequency in the BM and increased frequency in the PB, indicative of a systemic immune response. Most notably, we observed a significant reduction in T cell frequencies, from 64.8% to 53.5% in PB and from 10.2% to 3.1% in BM, as PIS progresses. This decline in T cells is particularly striking and contrasts with the patterns observed in other immune subsets, underscoring the profound impact of sepsis on T cell populations. Among these subpopulations, *Arg2* transcripts were predominantly localized within myeloid cells, with expression levels of 10% in PB and 4% in BM myeloid cells (Figure S2, H and I).

**Figure 3:**
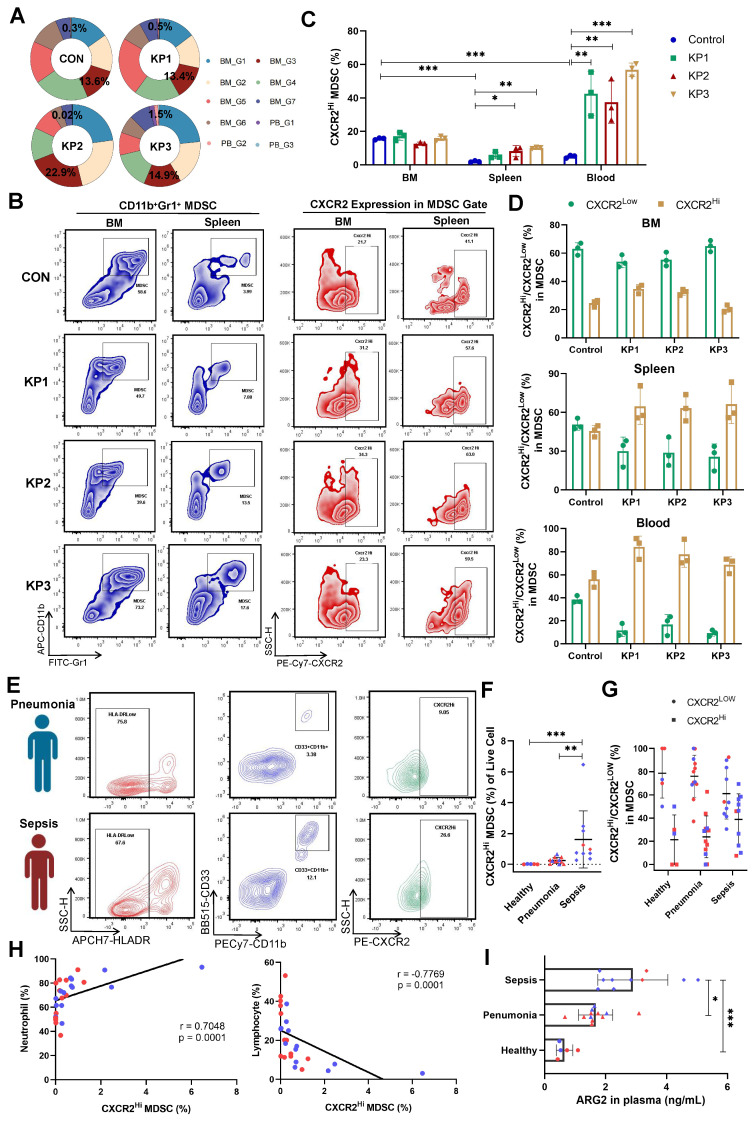
**Increase of ARG2-enriched CXCR2^Hi^ MDSC was negatively correlated with lymphocytes. (A)** The ring chart for relative proportions of granulocyte subclusters in PB and BM at CON, KP1, KP2, and KP3 stages. **(B)** Representative flow cytometry plots of the CD11b^+^Gr1^+^ MDSC and CXCR2^Hi^ subset in immune organs BM and spleen. **(C)** During PIS, proportions of ARG2-enriched CXCR2^Hi^ MDSCs increased in the spleen and PB. n = 3 biologically independent mice. Statistical significance was calculated by the Brown-Forsythe and Welch analysis of variance (ANOVA) test. **(D)** The CXCR2^Hi^ subset but not the CXCR2^Low^ subset dominated MDSCs in peripheral blood in PIS models. **(E)** Representative flow cytometry plots of the HLA-DR^Low^CD11b^+^CD33^+^ MDSC and CXCR2^Hi^ subset in blood samples from clinical patients. **(F)** Statistics of proportions of CXCR2^Hi^ MDSC in living cells among healthy volunteers (n = 5), pneumonia patients (n = 12), and septic patients (n = 10), as analyzed by one-way ANOVA analysis with nonparametric Kruskal-Wallis test.** (G)** With sepsis progression, the CXCR2^Hi^ subset expanded in MDSCs. **(H)** Correlations between the percentage of CXCR2^Hi^ MDSC and neutrophil proportion as well as lymphocyte proportion. **(I)** The ARG2 contents in plasma from the healthy (n = 5), pneumonia (n = 12), and septic patients (n = 10). **(F-I)** Red and blue dots represented female and male individuals, respectively. Statistical significance was calculated via one-way ANOVA analysis with nonparametric Kruskal-Wallis test. Data were presented as mean ± SD. *p < 0.05, **p < 0.01, ***p < 0.001.

Next, we re-clustered myeloid cells into monocytes, granulocytes, and dendritic cell subsets based on their distinct marker genes (Figure 2, B and C, Figure S2, J-K). At the single-cell resolution, dominant expression of *Arg2* was observed in granulocyte subtypes (PB_G2 and BM_G3), with expressing percentages at 30% and 40%, respectively (Figure 2, B and C). The expression of *Arg2* in lung tissues was further validated using a public dataset from septic mice induced by intratracheal *K.p* or LPS inoculation (Table S3). As a result, *Arg2* was primarily expressed in neutrophils (Figure S3, A, B, D, and E). These *Arg2*-expressing neutrophils accumulated upon intratracheal administration of LPS or bacteria (Figure S3, C and F). Integrating PB_G2 and BM_G3 profiles, we identified the *Arg2*-enriched granulocyte subset as myeloid-derived suppressor cells (MDSCs), characterized by the specific expression of MDSC markers *Cd11b* (*Itgam*) and *Gr1* (*Ly6g*) 33 (Figure 3E). Functional class scoring revealed that the MDSC score 34 was significantly upregulated in this subset, while other function scores related to bactericidal activities^25^, including NADPH oxidase complex and granular protein synthesis (specific granules and azurophil granules), were comparatively lower (Figure 3D and Table S4). Differential gene expression analysis using COSG 35 and FindMarker 26 identified high expression of CXCR2, along with 42 other signature DEGs, in the *Arg2*-enriched MDSCs (Figure 2, F-H and Table S5). Notably, the transcriptomic signature of this subset included multiple MDSC-associated molecules such as *Cd33*, *Cd84*,* Cd300ld*, *Acod1*, *Hdc*, and *Il1b* 34, 36-38.

The CXCR2 has been reported to be a marker for tumor-derived MDSCs 39. Given the specific and high expression of CXCR2, we identified it as a discriminating surface marker for isolating the targeted cell subset. Utilizing the FACS approach, we successfully separated CXCR2^Low^ and CXCR2^Hi^ MDSCs from BM samples of PIS and control mice, respectively (Figure 2I). Quantitative PCR analysis exhibited higher expression of signature genes (including *Ifitm1*, *Clec4d*, *Acod1*, *Arg2*, *Cxcl2*, *Ccl6*, *Cxcr2*, *Slc7a11*, *Csf3r*, *Cd84*, *Clec4e*, *Wfdc17*, *Lilr4b*, *Mxd1*, *Mmp9*, *Mmp8*, *Hdc*, *Cd33*, and *Il1b*) in the CXCR2^Hi^ MDSCs (control group in Figure 2J and PIS group in Figure S3G), aligning with the expression profile of *Arg2*-enriched granulocytes. In addition, the protein levels of ARG2 were notably elevated in the CXCR2^Hi^ MDSCs compared to the CXCR2^Low^ MDSCs (Figure 2K) in both PIS and control groups. These data confirmed that* Arg2* was enriched in CXCR2^Hi^ MDSCs.

### An increase of ARG2-enriched CXCR2^Hi^ MDSC was negatively correlated with lymphocytes

In our sc-RNA seq data, the proportions of CXCR2^Hi^ MDSCs were significantly higher in BM than in PB cells. The proportion was 13.6% in BM and 0.3% in PB cells in the CON group (Figure 3A), suggesting the origin of CXCR2^Hi^ MDSC in BM. However, CXCR2^Hi^ MDSCs in PB significantly expanded during disease progression (from 0.3% to 1.5%), while in BM, they slightly changed (from 13.6% to 14.9%). We further verified the expression of CXCR2^Hi^ MDSCs in animal models and clinical cohorts. The Figure 3B showed representative pictures of CXCR2^Hi^ MDSCs in flow cytometry assays. In the CON group of mice models, the highest proportion of CXCR2^Hi^ MDSCs was observed in BM (15.67%, compared to 1.98% in the spleen and 5.0% in the blood) (Figure 3C). After *K.p* inoculation, in response to pathogenic and inflammatory stimuli 40, the CXCR2^Hi^ MDSCs expanded quickly (Figure 3C), becoming the dominant subset in MDSCs (Figure 3D) in peripheral blood and spleen. At the same time, BM had a relatively stable CXCR2^Hi^ MDSC proportion, probably due to the release into the periphery following emergency granulopoiesis.

We further validated the expansion of CXCR2^Hi^ MDSCs in septic patients. A total of 27 individuals were enrolled, including 10 sepsis patients, 12 pneumonia patients as disease control, and five healthy controls. Demographic characteristics were detailed in Table S6. No significant differences were observed in gender and age. Septic patients suffered pronounced lymphopenia, with a median lymphocyte count of 0.93 × 10^9^ /L. The human MDSCs in circulation were marked by HLA-DR^Low^CD11b^+^CD33^+^ (Figure 3E and Figure S4A) 41. In septic patients, the MDSCs were significantly elevated when compared to those in healthy controls (3.44% vs. 0.01%, *p* < 0.01) (Figure S4B). Notably, the CXCR2^Hi^ MDSC, with higher ARG2 expression than the CXCR2^Low^ subset (Figure S4C), exhibited a pronounced increase in the sepsis group, as opposed to the healthy and pneumonia groups (0.85% vs. 0.23% and 0.01%, respectively, both *p* < 0.01) (Figure 3F). As sepsis advanced, the CXCR2^Hi^ subset showed a trend towards further expansion within the MDSC population (Figure 3G). The proportion of CXCR2^Hi^ MDSCs was positively correlated with the percentage of neutrophils (r = 0.7048, *p* < 0.01) and negatively correlated with the percentage of lymphocytes (r = -0.7769, *p* < 0.01) in the peripheral blood (Figure 3H and Figure S4, D-F). Consistent with the accumulation of CXCR2^Hi^ MDSCs, ARG2 contents in blood samples from septic patients were significantly elevated compared to either the healthy (2.89 ng/mL vs. 0.65 ng/mL, *p* < 0.001) or the pneumonia (2.89 ng/mL vs. 1.67 ng/mL, *p* = 0.015) group (Figure 3I and Figure S4G).

In addition, we conducted a comparative analysis to characterize the ARG2-enriched CXCR2^Hi^ MDSC phenotype in public blood transcriptome data of septic patients (Table S3). The analysis revealed a generally high expression of signature genes associated with this MDSC subset in neutrophils but not monocytes (Figure S4H), disclosing the granulocytic origin of CXCR2^Hi^ MDSCs. Furthermore, we utilized the receiver operating characteristic (ROC) analysis to assess the diagnostic potential of *Arg2* expression in distinguishing sepsis from healthy states, yielding an AUC range of 0.755 to 0.905 (Figure S4I). Additionally, through univariate regression analysis, we identified high *Arg2* expression as a risk factor for 28-day mortality in septic patients with prognostic information (OR > 1, *p* < 0.05) (Figure S4J). Together, these data indicated that the gene-phenotype of ARG2 enriched CXCR2^Hi^ MDSC derived from our PIS model was translatable into human disease and raised the hypothesis that the immunosuppressive activity burden by this MDSC subset might contribute to lymphopenia and subsequent poor prognosis in septic patients.

### CXCR2^Hi^ MDSC suppressed the proliferation of CD4^+^ T cells via ARG2-arginase activities* in vitro*

**Figure 4:**
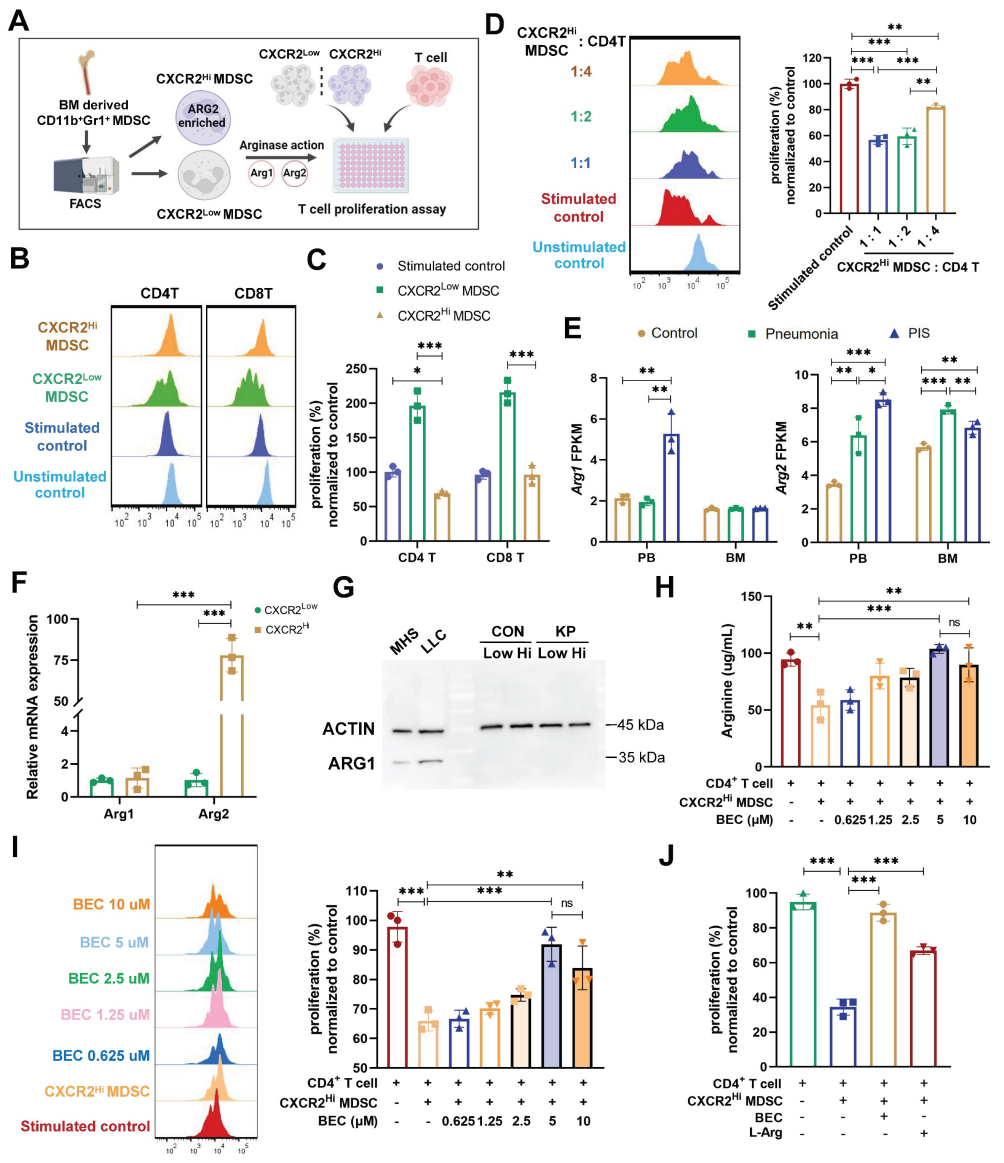
** The CXCR2^Hi^ MDSC suppressed the proliferation of CD4^+^ T cells depending upon ARG2-arginase activities *in vitro*. (A)** Experiment design to compare immunosuppressive functions of CXCR2^Low^ and CXCR2^Hi^ MDSC subsets. **(B)** Histogram overly exhibited CD4^+^/CD8^+^ T cell proliferation labeled by CFSE, as measured by flow cytometry. The ratios of co-culture numbers of MDSC and T cells were 1:1. **(C)** Quantitative bar charts showed the percentage of CD4^+^ and CD8^+^ T cell proliferation normalized to stimulated control. **(D)** The normalized percentage of CD4^+^ T cell proliferation at co-culture numbers of MDSC and T cells were 1:1, 1:2, and 1:4. **(E)** The abundance of *Arg1* and *Arg2* transcripts in PB and BM by bulk-RNA sequencing. **(F)** RT-PCR assay revealed remarkably high *Arg2* expression and low *Arg1* expression in CXCR2^Hi^ MDSC. Statistical significance was analyzed by unpaired t-test. **(G)** Western blotting assay showed extremely low protein content of ARG1 in both CXCR2^Low^ and CXCR2^Hi^ MDSC. Cell lines MH-S (mouse alveolar macrophage) and LLC (mouse Lewis lung cancer cell) were positive controls. **(H and I)** The **H** arginine concentration and **I** normalized percentage of CD4^+^ T cell proliferation elevated with BEC concentration increasing. The ratio of co-culture numbers of MDSC and T cells was 1:1. **(J)** Quantitative bar charts showing the normalized percentage of CD4^+^ T cell proliferation with treatment of BEC (10 μM) and L-Argine (100 μM). Statistical significances were calculated using the Brown-Forsythe and Welch analysis of variance (ANOVA) test unless otherwise indicated. Data were presented as mean ± SD. *p < 0.05, **p < 0.01, ***p < 0.001.

Studies have implicated MDSCs in suppressing T cell proliferation during sepsis 42. Our data revealed a negative correlation between ARG2-enriched CXCR2^Hi^ MDSCs and lymphopenia, suggesting a potential role of these cells in septic lymphopenia. To explore this relationship, we co-cultured BM-derived CXCR2^Hi^ MDSCs with splenic T lymphocytes and assessed their impact on CD4^+^ and CD8^+^ T cell proliferation (Figure 4A). Upon CD3/CD28 co-stimulation, CXCR2^Hi^ MDSCs exhibited a more pronounced suppressive effect on the proliferation of CD4^+^ T compared to CXCR2^Low^ MDSCs at various co-culture ratios (Figure 4, B and C), the proliferation rate was 69.15% vs. 196.4% (*p* < 0.001). And the immunosuppressive effect of CXCR2^Hi^ MDSC was diminished when its number in the co-culture was decreased (from 1:1 to 1:4), with a proliferation rate of CD4^+^ T cells increasing from 56.67% to 81.86% (*p* < 0.001) (Figure 4D). In contrast, no significant inhibitory effect was observed for CD8+ T cell proliferation (Figure 4, B and C). This might be attributed to discrepancies in expression levels of arginine transporters and intracellular arginine sensors (Figure S5, A and B).

Arginase activities mediated by isozymes ARG1 or ARG2 were required for MDSC to metabolize L-arginine to inhibit T-cell proliferation 43, 44. To determine the contribution of ARG1 and ARG2 to this effect, we first compared their mRNA levels in our sc-RNA seq data. As shown in Figure 4E, *Arg1* was elevated in PB but not in BM during PIS, whereas *Arg2* was significantly upregulated in both PB and BM. The increased *Arg2* expression in the PIS group compared to the pneumonia group within BM DEGs suggests that the systemic inflammatory response in sepsis enhances the release and peripheral migration of CXCR2^Hi^ MDSCs, which might amplify ARG2-mediated immunosuppression. Further validation confirmed the minimal expression of ARG1 in BM-derived MDSCs, irrespective of CXCR2^Hi^ or CXCR2^Low^ subsets (Figure 4F and 4G). The transcript level of *Arg2* was about 75-fold higher than that of *Arg1* in CXCR2^Hi^ MDSCs (*p* < 0.001). The above data confirmed that ARG2 but not ARG1 was enriched in the CXCR2^Hi^ MDSCs.

To substantiate the role of ARG2 in suppressing T cell proliferation, we investigated its enzymatic activity within the metabolic pathway of L-arginine. We assessed the* in vitro* capacity of CXCR2^Hi^ MDSCs to deplete arginine, a critical component for T cell proliferation. Notably, the co-culture of CXCR2^Hi^ MDSCs with CD4^+^ T cells led to a significant reduction in arginine levels in the supernatant (94.39 ug/mL vs. 54.17 ug/mL, *p* < 0.001), indicative of ARG2's metabolic activity (Figure 4H). To directly implicate ARG2 in this process, we incorporated the specific ARG2 inhibitor, BEC hydrochloride (BEC) 45, into the co-culture system. The incremental addition of BEC, ranging from 0.625 μM to 5 μM, correspondingly resulted in a dose-dependent restoration of arginine concentrations, from 58.72 ug/mL to 103.8 ug/mL (Figure 4H). This biochemical rescue was paralleled by a significant recovery in the proliferation of CD4^+^ T cells (65.96% vs. 91.91%, *p* < 0.001) (Figure 4I). Moreover, the exogenous supplementation of L-arginine to the co-cultures negated the suppressive effect of CXCR2^Hi^ MDSCs, further confirming the dependency of T cell proliferation on arginine availability (Figure 4J). In addition, although MDSCs were reported to inhibit the T-cell proliferation via PD-L1 in septic models, PD-L1 was remarkably lower expressed in CXCR2^Hi^ MDSCs than in the CXCR2^Low^ MDSCs (Figure S6A), indicating different mechanisms underlying heterogeneous MDSC subtypes. These results underscored that ARG2, but not ARG1, is predominantly expressed in CXCR2^Hi^ MDSCs and is pivotal for the observed inhibition of T-cell proliferation through the L-arginine metabolic pathway.

### BEC-mediated ARG2 inhibition regained CD4^+^ T cells and improved host defense against secondary infection *in vivo*

The influence of ARG2 inhibition on CD4^+^ T cell functionality* in vivo* was further explored to delineate its role in sepsis immunopathology. Initially, the survival rates were compared between BEC-treated and control mice in our lethal PIS models, with or without levofloxacin. However, no significant improvement was observed among the various groups (Figure S6, B and E). This might be due to BEC's inability to mitigate the early inflammatory cytokine release (Figure S6, C and D), a primary cause of mortality in septic hosts.

**Figure 5:**
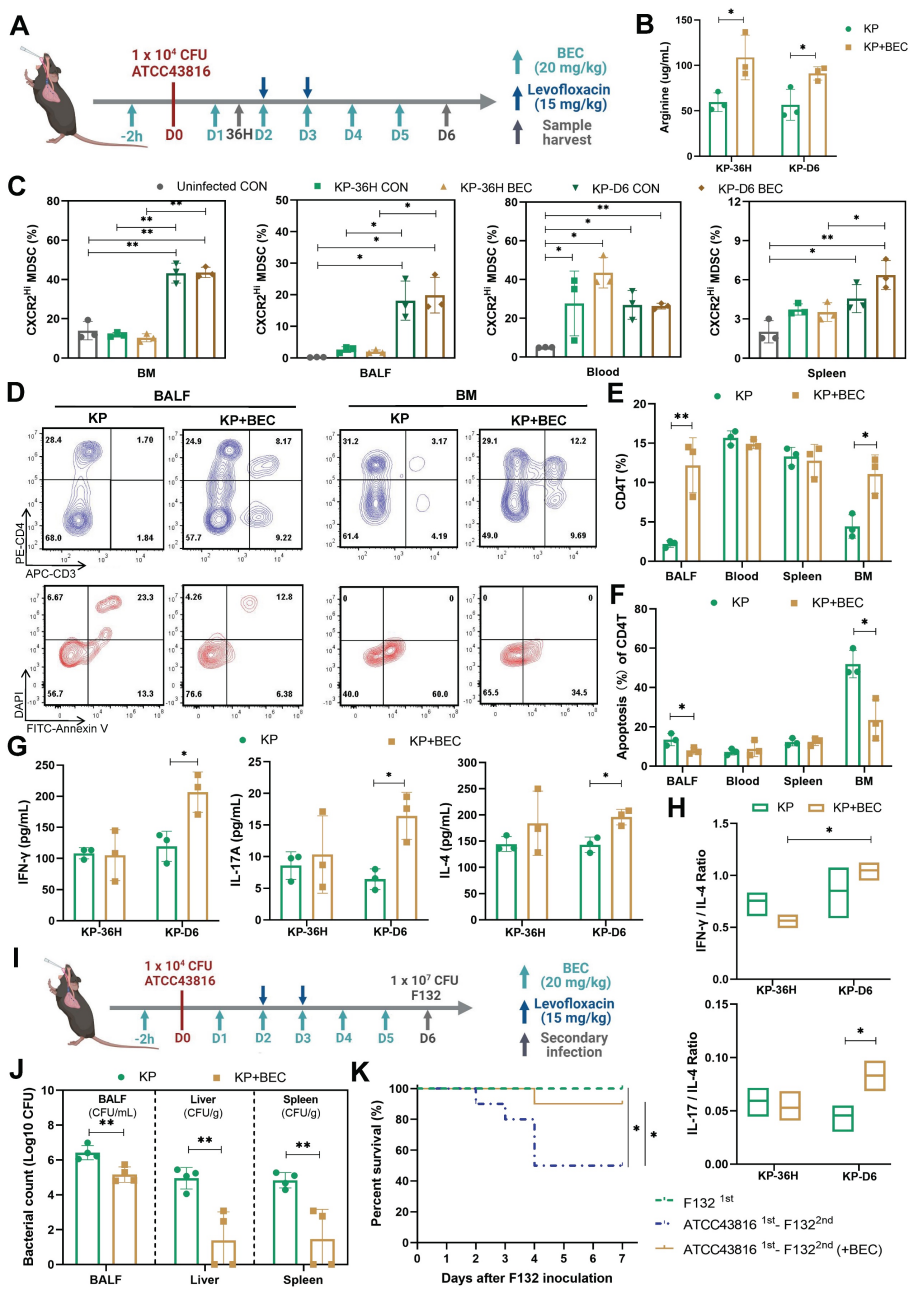
** ARG2 inhibitor BEC regained CD4^+^ T cells and improved host defense against secondary infection. (A)** Schematic diagram of the BEC administration and sample collection procedures at 36 h (36H) and 6 days (D6) based on PIS models. **(B)** BEC administration restored arginine in bronchoalveolar lavage fluid (BALF). **(C)** The dynamic detection of CXCR2^Hi^ MDSCs in BALF, BM, spleen, and blood showed their accumulation in BALF and BM at D6, as calculated by Brown-Forsythe and Welch analysis of variance (ANOVA) test. n = 3 biologically independent mice. **(D)** Representative flow cytometry plots of CD4^+^ T cell and its apoptosis in BALF and BM at D6. **(E and F)** Elevated CD4^+^ T cell percentage **E** and reduced apoptotic rate **F** in BALF and BM in the KP+BEC group at D6. n = 3 biologically independent mice. **(G)** Increased levels of Th1-secreted cytokines IFN-γ, Th17-secreted IL17A, and Th2-secreted IL-4 in BALF at D6. n = 3 biologically independent mice. H) The elevation of IL-17A/IL-4 ratio in the KP+BEC group, indicating an enhanced CD4-T-cell response dominated by Th17, as analyzed by ANOVA test. **(I)** Schematic diagram of the first infection (strain #ATCC43816) and second hit (strain #F132) procedures in animal experiments to simulate secondary infection following sepsis in the clinical scenario. **(J)** Viable bacterial CFUs recovered from BALF, liver, and spleen after 24 h of secondary infection. n = 4 biologically independent mice. **(K)** Kaplain-Meier survival curve of mice undergoing first infection with #F132, and second hit with #F132 following first infection with #ATCC43816 when giving BEC or not (n = 10 mice/group). Animal data were all from female c57BL/6 mice. Statistical significances were calculated by unpaired t-test unless otherwise indicated. Data were presented as mean ± SD. *p < 0.05, **p < 0.01.

Decreased CD4^+^ T cell count was related to chronic immunoparalysis and increased susceptibility to infections 11, 46. Thus, we investigated the effects of BEC on PIS at a later stage and its influence on reactivity to secondary infections. BEC was administered intraperitoneally at 20 mg/(kg·d) to evaluate its impact on T cell immune dysfunction post-PIS induction (Figure 5A). BEC significantly elevated arginine levels in BALF at 36 h and D6 post-infection (108.8 ug/mL vs. 59.55 ug/mL, *p* = 0.033; 91.22 ug/mL vs. 56.67 ug/mL, *p* = 0.032, respectively) (Figure 5B), without affecting plasma levels (Figure S6F). The ARG2-enriched CXCR2^Hi^ MDSCs expanded systemically at a later stage (D6), including BM, blood, BALF, and spleen, but only expanded in the peripheral blood at 36 h post-infection (Figure 5C). Similar findings could be observed in cecum ligation and puncture (CLP) models simulating the abdominal sepsis. We first confirmed that the *Arg2* transcripts was indeed enriched in the CXCR2^Hi^ MDSC subset under CLP states via sc-RNA analysis (Figure S7, A-D). Subsequently, utilizing the surviving CLP model with imipenem treatment 47 (Figure S7E), we demonstrated that CXCR2^Hi^ MDSCs also accumulated systemically at a later stage (D6) after surgery, and their proportions in BM, BALF, blood were significantly elevated in the CLP group than the Sham group (Figure S7F).

Notably, despite no reduction in ARG2-enriched CXCR2^Hi^ MDSCs numbers post-BEC, a substantial decrease in CD4^+^ T cell apoptosis was observed in BALF (7.7% vs. 13.5%, *p* = 0.046) and BM (23.46% vs. 51.93%, *p* = 0.017) on day 6 post-infection, effectively restoring CD4+ T cell proportions from 2.18% to 12.19% (*p* = 0.008) in BALF and from 4.42% to 11.07% (*p* = 0.016) in BM (Figure 5, D-F, and Figure S6, G and H). Immunofluorescence analysis of lung tissues also confirmed increased CD4^+^ T cell infiltrations (*p* = 0.008) in BEC-treated mice compared to infected controls (Figure S6I). Additionally, BEC treatment was associated with increased levels of CD4^+^ T cell effectors cytokines, including IFN-γ (206.5 pg/mL vs. 119.6 pg/mL, *p* = 0.021), IL-17A (16.4 pg/mL vs. 6.4 pg/mL, *p* = 0.013), and IL-4 (196.0 pg/mL vs. 143.1 pg/mL, *p* =0.012), as well as an elevation of the IL-17A to IL-4 ratio (*p* = 0.043) (Figure 5, G and H), suggesting a shift towards a Th17 response, which is crucial for airway bacterial clearance during the memory response 48.

Furthermore, we investigated the impact of BEC treatment on susceptibility to secondary infections. Using a clinical *K.p* strain (F132) 22, which was isolated from a nosocomial infection in the ICU, we induced secondary pneumonia in mice recovering from PIS. BEC treatment significantly reduced the bacterial loads in BALF and distal organs, such as the liver (3.56-log lower CFUs, *p* = 0.006) and spleen (3.30-log lower CFUs, *p* = 0.006), leading to improved survival rates (90% vs. 50%, *p* = 0.048) in the two-hit model (Figure 5, J and K). These findings demonstrate that targeting ARG2-enriched CXCR2^Hi^ MDSCs with BEC ameliorates T-cell dysfunction and enhances the host's resistance to secondary infections following PIS.

### The ARG2 knockdown in ARG2-enriched CXCR2^Hi^ MDSCs were favorable for CD4^+^ T cell and host's immune response against secondary infection

**Figure 6:**
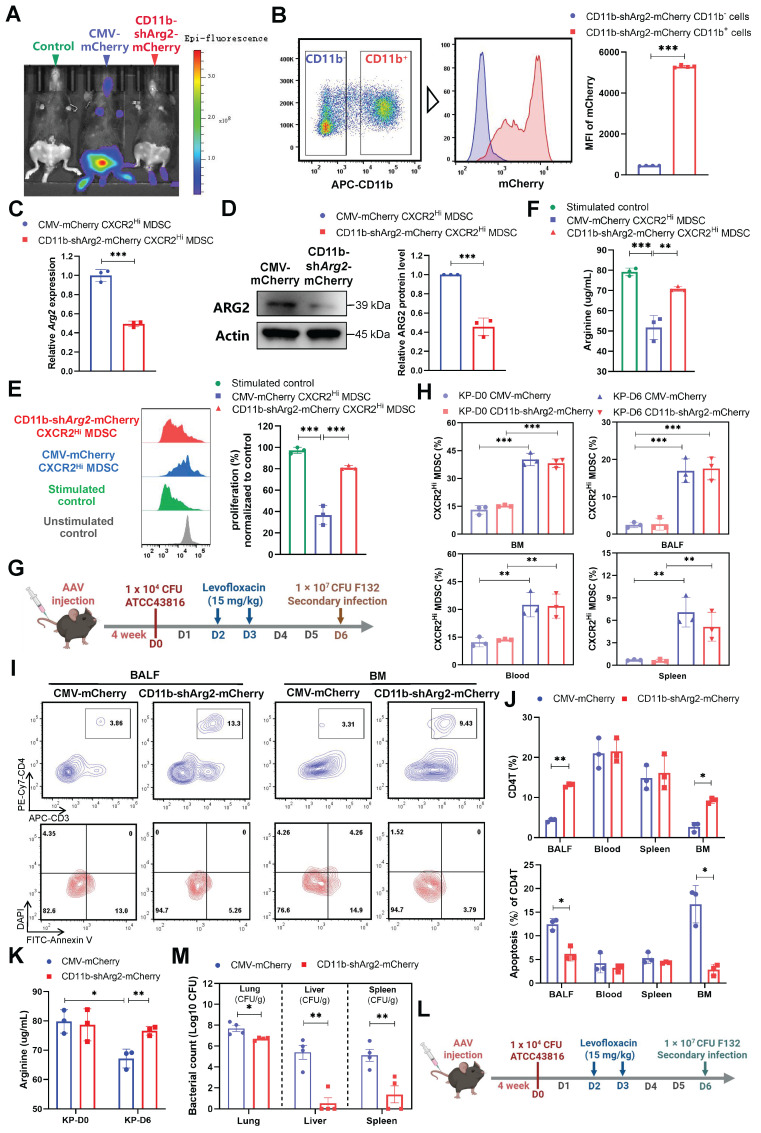
** The CD11b-specific ARG2 knockdown were favorable for CD4^+^ T cell proliferation and survival. (A)** *In vivo* fluorescence (mCherry) image 4 weeks after AAV infection confirmed the expression of AAV genes. **(B)** The CD11b^+^ cells from CD11b-shArg2-mCherry mice showed intense mCherry fluorescence, confirming the expression specificity of the CD11b promoter. n = 4 biologically independent mice. **(C and D)** Validation of ARG2 knockdown in primary CXCR2^Hi^ MDSC cells via **C** RT-PCR and **D** western blotting assays. Data are representative of one replicate of three independent experiments. **(E and F)** The **E** proliferation ratio of CD4^+^ T cell and **F** supernatant arginine concentration were recovered when co-cultured with CXCR2^Hi^ MDSC from CD11b-shArg2-mCherry mice. The ratios of co-culture numbers of MDSC and CD4^+^ T cells were 1:2. Statistical significances were calculated by Brown-Forsythe and Welch analysis of variance (ANOVA) test.** (G)** Schematic representation of PIS model induction and sampling 4 weeks after AAVs injection.** (H)** The proportion of CXCR2^Hi^ MDSCs in BALF, BM, spleen, and blood in CMV-mCherry and CD11b-shArg2-mCherry group at D0 and D6. n = 3 biologically independent mice. **(I)** Representative flow cytometry plots of CD4^+^ T cell and its apoptosis in bronchoalveolar lavage fluid (BALF) and BM at D6. **(J)** Elevated CD4^+^ T cell percentage and reduced apoptotic rate in BALF and BM in the CD11b-shArg2-mCherry group at D6. n = 3 biologically independent mice. **(K)** The ARG2 knockdown restored depleted arginine in BALF.** (L)** Schematic representation of the first infection (strain #ATCC43816) and second hit (strain #F132) procedures in animal experiments 4 weeks after AAVs injection.** (M)** Viable bacterial CFUs recovered from lung, liver, and spleen in AAV-delivered mice after 24 h of secondary infection. n = 4 biologically independent mice. Animal data were all from female c57BL/6 mice. Statistical significances were calculated by unpaired t-test unless otherwise indicated. Data were presented as mean ± SD. *p < 0.05, **p < 0.01, ***p < 0.001.

To further determine whether the downregulation of CXCR2^Hi^-MDSC-derived ARG2 exerted preventive effects against septic immunosuppression. We delivered the adeno-associated virus (AAV) 2/8 with CD11b promoter and short hairpin RNA sequences into mice via tail vein injection, to achieve the specific knockdown of ARG2 in CD11b^+^ myeloid cells (including MDSCs marked by CD11b^+^Gr1^+^) 49. Four weeks after AAV injection, we noted the expression of AAV genes *in vivo*, as indicated by the fluorescence of mCherry (Figure 6A). In contrast to the general expression of the AAV control CMV-Cherry, the AAV CD11b-shArg2-mCherry was selectively expressed in the BM, the ecological niche of myeloid cells. This expression specificity was better confirmed via flow cytometry, where the fluorescence intensity of mCherry from BM-derived CD11b^+^ cells was 11.75-fold higher (*p* < 0.001) than that of CD11b^-^ cells in CD11b-shArg2-mCherry mice (Figure 6B). Compared to the CMV-mCherry group, CXCR2^Hi^ MDSCs isolated from CD11b-shArg2-mCherry mice showed an approximately 50% decrease in both mRNA and protein levels of ARG2 (both *p* < 0.001) (Figure 6, C and D).

To corroborate the role of CXCR2^Hi^ MDSC-derived ARG2 in inhibiting T cell proliferation, we performed *in vitro* co-culture experiments with ARG2-knockdown CXCR2^Hi^ MDSCs and CD4^+^ T cells. As shown in Figure 6E, CXCR2^Hi^ MDSCs isolated from CMV-mCherry mice harbored the same suppressive capacity on CD4^+^ T cell proliferation as before. However, this capacity was substantially impaired in ARG2-knockdown CXCR2^Hi^ MDSCs isolated from CD11b-shArg2-mCherry mice, as illustrated by the significant recovery in the proliferation of CD4^+^ T cells (81.16% vs. 36.64%, *p* < 0.001). Meanwhile, in the co-culture system of ARG2- knockdown CXCR2^Hi^ MDSCs, the arginine necessary for T-cell activation and proliferation was simultaneously rebounded (70.86 ug/mL vs. 51.76 ug/mL, *p* = 0.001) (Figure 6F).

We then carried out *in vivo* experiments to investigate the impact of CD11b-specific ARG2 knockdown on CXCR2^Hi^ MDSC and CD4^+^ T cells in PIS models (Figure 6G). Consistently, the CXCR2^Hi^ MDSCs accumulated in BM, blood, BALF, and spleen at a later stage (D6), which was not influenced by the inhibition or knockdown of ARG2 (Figure 5C and Figure 6H). As for CD4^+^ T cells, at day 6 post-infection, a remarkable decrease in CD4^+^ T cell apoptosis was noted in BALF (6.17% vs. 12.47%, *p* = 0.004) and BM (2.82% vs. 16.69%, *p* = 0.004) in CD11b-shArg2-mCherry mice, enabling increased CD4^+^ T cell proportions from 4.33% to 13.10% (*p* < 0.001) in BALF and from 2.66% to 9.24% (*p* = 0.001) in BM (Figure 6, I and J). Additionally, PIS induction led to significantly reduced arginine levels in BALF (67.15 ug/ ug/mL vs. 79.83 ug/mL, *p* = 0.013) on day 6 (Figure 6K), whereas the ARG2 knockdown in CD11b-shArg2-mCherry group rescued arginine depletion (76.66 ug/mL vs. 67.15 ug/mL, *p* = 0.01), in line with the role of ARG2 as an arginase in *in vitro* experiments. Moreover, in the two-hit experiment induced by the opportunistic strain F132 (Figure 6L), the bacterial load in lung tissues (1.0-log lower CFUs, *p* = 0.017) as well as distal organs liver (3.30-log lower CFUs, *p* = 0.001) and spleen (3.30-log lower CFUs, *p* = 0.009) of mice in CD11b-shArg2-mCherry group was significantly lowered in comparison to the CMV-mCherry group (Figure 6M), indicating a reduced susceptibility to secondary infections. Overall, our data clearly showed that both the pharmacological inhibition and knockdown of ARG2 in ARG2-enriched CXCR2^Hi^ MDSCs were favorable for CD4^+^ T cell proliferation and survival, thereby improving host's immune response against opportunistic bacteria.

### The p38-MAPK pathway as a central regulator of ARG2-enriched CXCR2^Hi^ MDSCs in PIS

**Figure 7:**
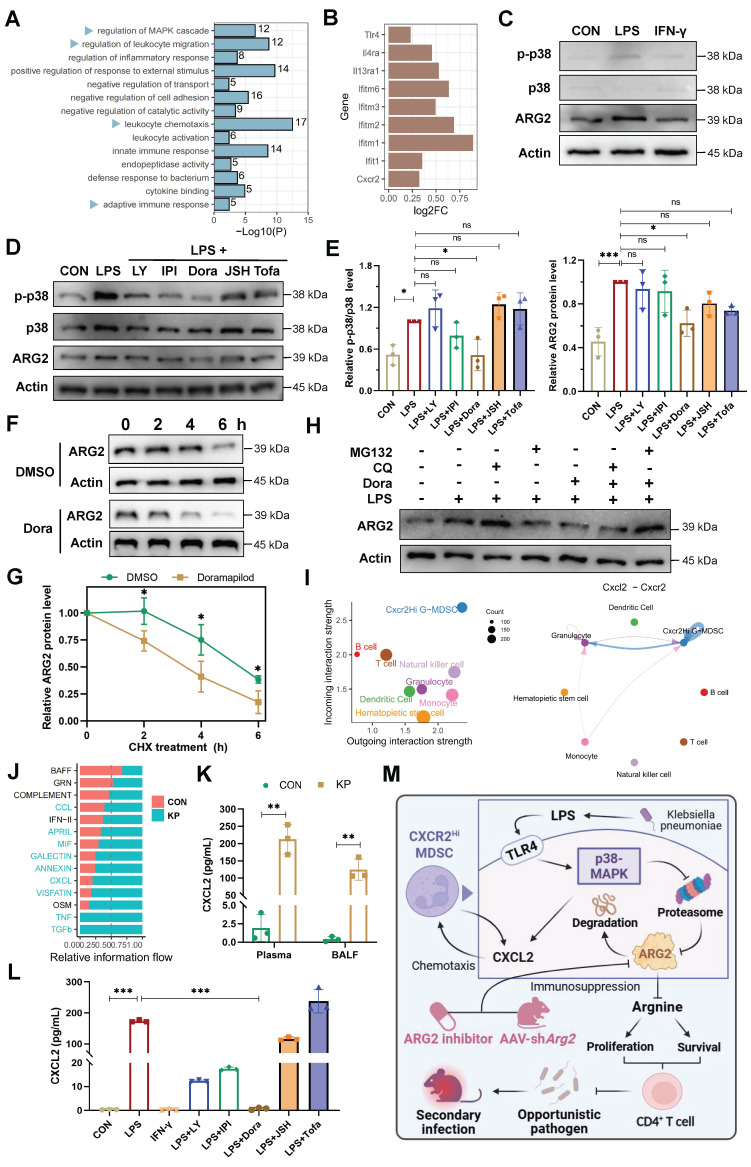
** The p38-MAPK pathway as a central regulator of ARG2-enriched CXCR2^Hi^ MDSC in PIS. (A)** Gene ontology (GO) terms dominated in the CXCR2^Hi^ MDSC, where the MAPK cascade was significantly enriched. **(B)** The transcript levels of *Tlr4*, *Il4ra*, *Il13ra1*, *Cxcr2*, and Interferon-stimulated genes (ISGs) upregulated upon infection.** (C)** LPS induced the upregulation of ARG2, along with activation of p38-MAPK signaling. **(D)** The pretreatment of p38 inhibitor Doramapimod impeded the upregulation of ARG2 and p38-MAPK pathway activity when LPS stimulation. LY: The PI3Kα, PI3Kδ, and PI3Kβ inhibitor LY294002. IPI: The PI3Kγ inhibitor IPI594. Dora: The p38-MAPK inhibitor Doramapimod. JSH: The NF-κB inhibitor JSH-23. Tofa: The JAK inhibitor Tofacitinib. **(E)** Quantitative bar graphs showed that the intervention of p38-MAPK inhibitor Dora significantly reduced p-p38/p38 and ARG2 protein levels compared with LPS treatment alone. **(F and G)** The half-life of ARG2 in isolated CXCR2^Hi^ MDSC when treated with the DMSO/Dora and cloheximide (CHX) for indicated times. Statistical significance was calculated via the unpaired t-test. **(H)** Inhibition of the proteasome via MG132 reversed ARG2 downregulation induced by the p38 inhibitor Dora. **(I)** Cellchat analysis revealed strong communication probabilities of Cxcl2-Cxcr2 chemotaxis between CXCR2^Hi^ MDSC and itself. **(J)** The bar plot showed relatively increased CXCL signaling flow at infected status.** (K)** Elevated CXCL2 levels in plasma and BALF samples from PIS mice models, as calculated by unpaired t-test. n = 3 biologically independent mice. **(L)** LPS stimulation resulted in increased secretion of CXCL2 by CXCR2^Hi^ MDSCs, which was reversed by the p38 inhibitor Doramapimod. Statistical significance was calculated by the Brown-Forsythe and Welch analysis of variance (ANOVA) test. **(M)** Proposed model demonstrating the p38-MAPK cascade regulated immunosuppressive function and migration of CXCR2^Hi^ MDSC in *K.p*-relevant PIS. The illustration was created by BioRender. Data were presented as mean ± SD. *p < 0.05, **p < 0.01, ***p < 0.001.

To decipher underlying regulatory signals regulating the functional activity of ARG2-enriched CXCR2^Hi^ MDSC, we conducted an enrichment analysis based on signature DEGs (Figure 2H). The result revealed significant enrichment of MAPK cascade signaling, cell migration processes, and immune response regulation pathways (Figure 7A). During sepsis, we observed up-regulation of surface receptors, including *Toll-like receptor 4* (*Tlr4*), *Interleukin-4 receptor alpha* (*Il4ra*), *Interleukin-13 receptor alpha-1* (*Il13ra1*), and interferon-stimulated genes (IFITM family members including *Ifitm6*, *Ifitm3*, *Ifitm2*, *Ifitm1*, and *Ifit1*), implying the involvement of LPS (*Tlr4* ligand), IL-4, IL-13, and interferon (IFN) in the immunomodulatory activities of this MDSC subset in septic hosts (Figure 7B).

Given that LPS and IFN-γ were major pathogenic factors in bacterial infections, we stimulated primary ARG2-enriched CXCR2^Hi^ MDSCs with LPS and IFN-γ in *vitro* to mimic their response to infection. LPS stimulation elicited a pronounced upregulation of ARG2 expression and increased p38 phosphorylation, indicating activation of the MAPK signaling pathway (Figure 7C). Notably, among various signaling pathway inhibitors (including PI3K, NF-κB, and JAK blockade), pretreatment with the p38-MAPK inhibitor Doramapimod (Dora) specifically abrogated the upregulation of ARG2 (*p* = 0.013) (Figure 7, D and E), highlighting the p38-MAPK cascade as a central regulator of the immunosuppressive function of CXCR2^Hi^ MDSCs through ARG2 engagement. We noted a discrepancy between the transcriptional regulation of *Arg2* and its protein levels upon p38-MAPK activation (Figure S8A), suggesting a post-transcriptional mechanism potentially involving protein degradation. To determine whether reduced ARG2 protein levels were caused by diminished synthesis or accelerated degradation, we treated cells with the translation-blocking agent cycloheximide (CHX) alongside p38 inhibitor. As shown in Figure 7F and 7G, in CXCR2^Hi^ MDSCs, the half-life of the ARG2 protein was shortened from 6 hours to 4 hours following treatment with the p38 inhibitor Dora. Furthermore, while the blockade of p38-MAPK signaling led to a marked reduction in ARG2 protein levels, the addition of the proteasome inhibitor MG132 rather than the autophagy inhibitor chloroquine (CQ) reversed this effect (Figure 7H). These results indicated that p38-MAPK signaling contributes to the stabilization of ARG2 protein by inhibiting its degradation via the proteasome.

In addition, using Cellchat analysis, we mapped the cell-cell communication network involving CXCR2^Hi^ MDSCs and other immune cell subpopulations. This analysis identified robust incoming and outgoing signals for CXCR2^Hi^ MDSCs, with the Cxcl-Cxcr chemotactic axis, primarily driven by the Cxcl2-Cxcr2 interaction, emerging as the most potent signal (Figure 7I and Figure S8, C-E). Following *K.p* infection, there was a substantial increase in the overall Cxcl signaling flow (Figure 7J), corroborated by significantly elevated levels of CXCL2 in plasma (212.6 pg/mL vs. 1.9 pg/mL, *p* = 0.001) and BALF (124.7 pg/mL vs. 0.4 pg/mL, *p* = 0.002) from PIS models (Figure 7K). Intriguingly, LPS stimulation of ARG2-enriched CXCR2^Hi^ MDSCs led to a consistent increase in CXCL2 transcripts and secretion (173.4 pg/mL vs. 0.4 pg/mL, *p* < 0.001) (Figure S8B and Figure 7L). These effects were then reversed entirely by Dora pretreatment (both *p* < 0.001), suggesting that the p38-MAPK regulated the CXCL2 at the transcriptional level. These findings suggested that the p38-MAPK pathway is a potential therapeutic target for modulating the immunosuppressive function and chemotactic behavior of ARG2-enriched CXCR2^Hi^ MDSCs in PIS (Figure 7M).

## Discussion

In the current study, we identify the pivotal role of ARG2 in the immunopathogenesis of PIS, highlighting the significance of ARG2-enriched CXCR2^Hi^ MDSCs in mediating T cell depletion through arginine starvation, a critical substrate for T cell proliferation. Our findings underscore the therapeutic potential of targeting ARG2, as evidenced by the restorative effects of ARG2 blockade and knockdown on CD4^+^ T cell counts and the subsequent enhancement of the host's defense against secondary infections. Furthermore, we identify the p38-MAPK pathway as a central regulator of ARG2-enriched CXCR2^Hi^ MDSCs, with its activation leading to increased ARG2 expression and CXCL2 production in response to bacterial infections. The targeted inhibition of p38-MAPK effectively counteracts these effects, demonstrating the pathway's role in controlling the immunosuppressive functions and chemotactic behavior of these MDSCs. This research advances the understanding of PIS immunopathology and paves the way for developing precision therapies to modulate the ARG2-enriched CXCR2^Hi^ MDSC to combat septic lymphopenia and immunosuppression.

We have uncovered a critical subpopulation of MDSCs, ARG2-enriched CXCR2^Hi^ MDSC, that are pivotal in the pathogenesis of septic lymphopenia. These cells, identified through single-cell transcriptome analysis, exhibit a distinct gene signature that sets them apart from standard myeloid cells, including the upregulation of immunomodulatory genes such as *Arg2*, *Cd84*, *Cd300ld*,* Acod1*, and *Hdc* 34, 36-38, as well as chemotactic genes such as *Cxcl2* and *Cxcr2*. This discovery provides a specific surface marker, CXCR2, in conjunction with CD11b/Gr1 for mice or HLA-DR/CD11b/CD33 for humans, which can be utilized for the diagnostic detection and functional study of MDSCs. The ARG2-enriched CXCR2^Hi^ MDSCs' ability to migrate to inflammatory sites through a CXCL2-CXCR2-mediated chemotactic mechanism mirrors the behavior of MDSCs in the tumor microenvironment, suggesting a commonality in their immunosuppressive behavior across different pathological contexts 50. Importantly, our data indicated a significant association between this MDSC subpopulation's peripheral frequency and lymphopenia's severity, highlighting its potential as a biomarker for immunological assessment in sepsis.

We revealed that the CXCR2^Hi^ MDSC subpopulation triggered apoptosis and impaired proliferation of CD4^+^ T cells in an ARG2-dependent way via arginine depletion. This finding aligns with the growing body of evidence supporting the potential of immunometabolic modulation as a therapeutic strategy for sepsis. L-arginine, a conditionally essential amino acid, has been shown to exert multiple beneficial effects on T cells, including promoting cell proliferation, activation, and survival 51. The starvation of L-arginine caused severe T-cell dysfunction. Increased arginine availability has been shown to support the restoration of T cell proliferative capacity and mitochondrial function, effectively decreasing apoptosis rates 52. Clinical research also confirmed that a deficiency state of arginine in septic patients has been associated with increased nosocomial infections and mortality 53. Thus, maintenance of arginine levels in septic patients is expected to improve clinical outcomes. However, direct arginine supplementation failed to improve the outcomes of critically ill hosts in both preclinical and clinical trials 52, 54. The pathophysiology of sepsis could be more complex than mere arginine deficiency. People further explored the therapeutic efficacies of targeting L-arginine metabolic enzymes. ARG1 and ARG2 are isoenzymes that catalyze L-arginine metabolism to L-ornithine and urea. Studies have reported that peripheral blood MDSCs in sepsis catalyzed arginine catabolism through the ARG1 activity 18, 55, 56. We found that the *Arg1* expression was elevated in PB but not in BM during PIS, this established the role of ARG1 in PB likely superseded any potential contribution from ARG2, partly explaining the lack of significant CXCR2^Hi^-MDSC-mediated effect on T cells in PB. In addition, studies have characterized the cumulative expression of ARG2 in lung myeloid cells in the severe COVID-19, thermal injury, and lung metastasis model of breast cancer to induce diminished immune surveillance 57-59, suggesting that the alveolar space could provide a permissive environment for ARG2-mediated immunosuppression. In our PIS models, the ARG2-enriched CXCR2^Hi^ MDSC functioned in the BM in addition to the lung, suggesting that a comparable situation may exist at the BM site. A global knockout of the ARG1 gene in mice led to severe hyperargininemia, neurological damage, and fatal hyperammonemia 60, 61. In contrast, besides a mild elevation of circulating levels of arginine, no severe abnormality was observed in ARG2 deficiency mice 62. Therefore, developing novel drugs or antibodies selectively targeting ARG2 offered an attractive immunotherapeutic method to prevent T-cell lymphopenia and improve the prognosis of critically ill patients.

The inhibition of MDSCs' immunosuppressive mechanisms has emerged as an effective strategy for reactivating T-cell responses and enhancing the efficacy of immunotherapies. Our study has identified the p38-MAPK signaling pathway as a critical regulatory axis controlling the tolerogenic activity of CXCR2^Hi^ MDSCs. This finding complements the established role of PI3Kγ in modulating immune suppression and inflammation of macrophages 63. Similar to the reported suppression of p38 signaling, which dismantles ARG1-mediated immunosuppression in MDSCs and potentiates T-cell antifungal activity against *Cryptococcus neoformans* 43, our findings suggested that targeting the p38-MAPK cascade with Doramapimod could be a viable therapeutic approach. By reversing the upregulation of ARG2 and CXCL2, Doramapimod has the potential to simultaneously curb immunosuppression and the Cxcl2-Cxcr2 chemotaxis, positioning it as a promising candidate for immunotherapeutic intervention in diseases characterized by MDSC-driven immune tolerance.

There were several limitations in our study. First, despite the valuable insights gained from our use of the primary MDSC cell, we are cognizant of its limitations in fully emulate the intricate physiological environment and complex interactions in the whole organism. This limitation underscores the need for future studies to incorporate conditional knockout mice models, which will allow us to delve deeper into the roles of ARG1 and ARG2 in septic immunity and provide a more holistic view of their functions. Second, whether and how the p38-MAPK regulates ARG2 protein through the molecular chaperone-mediated proteasome process deserve to be investigated in depth in subsequent researches. Third, whereas our design prioritizes mechanistic clarity in a controlled sex-specific context, we acknowledge that certain immune pathways may exhibit sex dimorphism 64. Future studies comparing sexes under standardized hormonal conditions will refine these distinctions.

## Conclusions

In conclusion, our research underscores the critical role of ARG2-enriched CXCR2^Hi^ MDSCs in sepsis-induced lymphopenia and immunosuppression and highlights the p38-MAPK pathway as a central regulator of CXCR2^Hi^ MDSCs. We also reveal the potential of ARG2 inhibition to restore CD4^+^ T cell and enhance resistance to secondary infections. These findings contribute to the growing understanding of the immunopathogenesis of sepsis and offer fresh horizons for immunotherapeutic intervention in sepsis.

## Supplementary Material

Supplementary figures and tables.

## Abbreviations

MDSC: Myeloid-derived suppressor cells
ARG2: arginase-2
ARG1: arginase-1
*K.p*: klebsiella pneumoniae
PIS: pneumonia-induced sepsis
sc-RNA seq: single cell-RNA sequencing
DEGs: Differentially expressed genes
LPS: lipopolysaccharide
PB: peripheral blood
BM: bone marrow
BALF: bronchoalveolar lavage fluid
FACS: fluorescence-activated cell sorting
PD-L1: programmed death 1 ligand
CXCR2: chemokine receptor 2
CXCL2: chemokine ligand 2
BEC: BEC hydrochloride
AAV: adeno-associated virus
shRNA: short hairpin RNA
PCA: principal component analysis
