# Supplementary material for: Pharmacological and genetic inhibition of ARG2 in CXCR2Hi myeloid-derived suppressor cells combats sepsis-induced lymphopenia
Source: Theranostics. 2025 Jul 11;15(16):7990–8011. doi: 10.7150/thno.112339 (PMC12374545; doi:10.7150/thno.112339)
Supplement: Supplementary file 1 — Supplementary figures and tables. [file thnov15p7990s1.pdf]

## Supplementary Figures

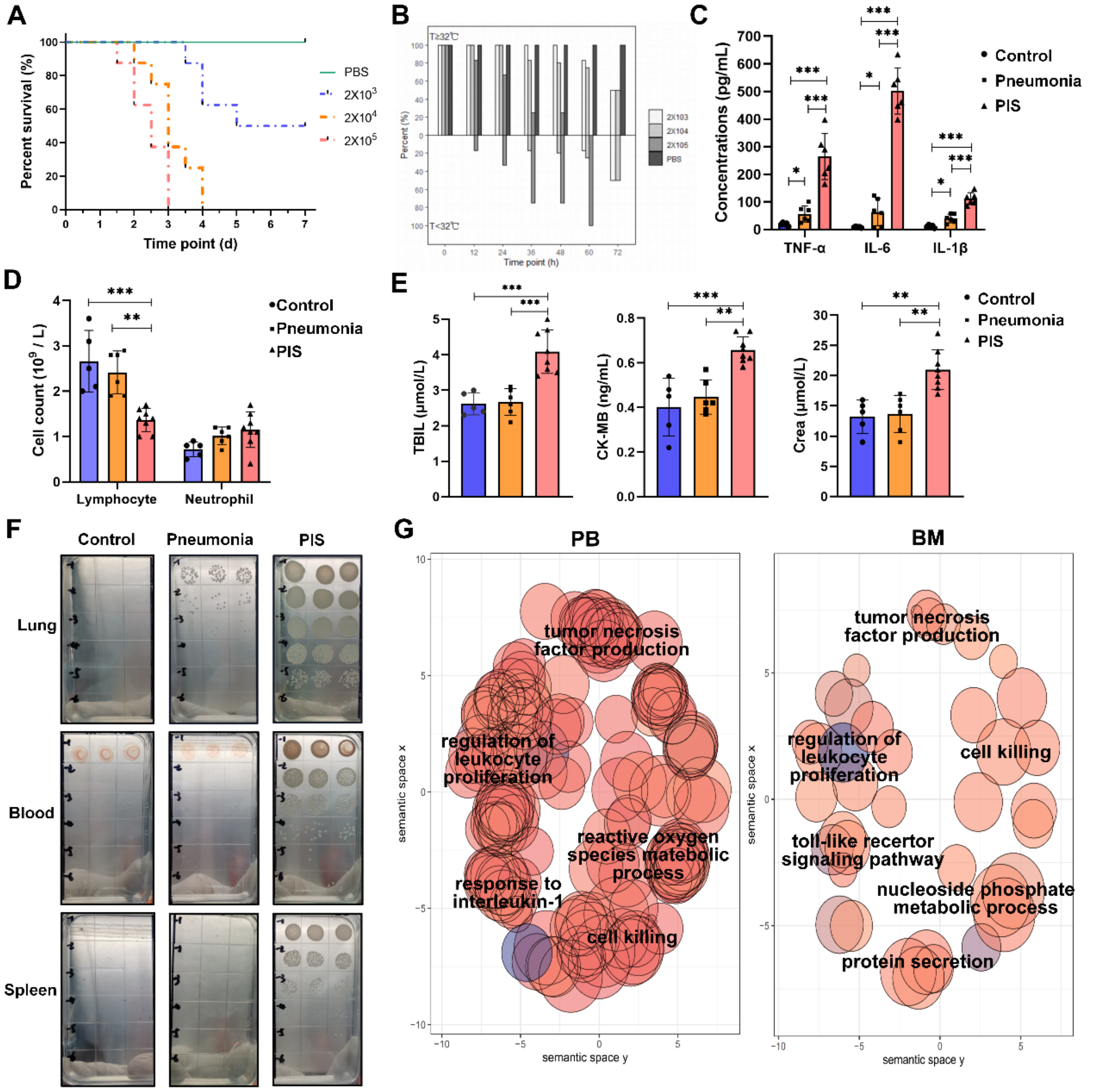


**Supplementary Figure 1. Supplementary data of *K.p*-induced sepsis model and bulk-RNA sequence.** Inoculation with PBS (control) and *K.p* ($2\times10^3$, $2\times10^4$, and $2\times10^5$ CFU), **(A)** the survival rate (n = 10 mice/group) and **(B)** low temperature (< 32 ℃) percent in each group. **(C)** Quantitative bar charts showing concentrations of TNF-α, IL-6, and IL-1β concentrations in BALF samples. **(D)** Quantitative bar charts showing lymphocyte and neutrophil counts in peripheral blood. **(E)** Comparisons of blood biochemical indexes indicating liver (TBIL), heart (CK-MB), and kidney (Crea) functions, as calculated by ANOVA. **(F)** Representative diaphragms of viable bacteria recovered from lung, blood, and spleen in control, pneumonia, and PIS groups. **(G)** Based on DEGs from PB and BM, the visualization of representative GO terms after eliminating redundancy with Revigo. Statistical significances were calculated using the Brown-Forsythe and Welch analysis of variance (ANOVA) test unless otherwise indicated. Data were presented as mean ± SD. *$p < 0.05$, **$p < 0.01$, ***$p < 0$ .001.

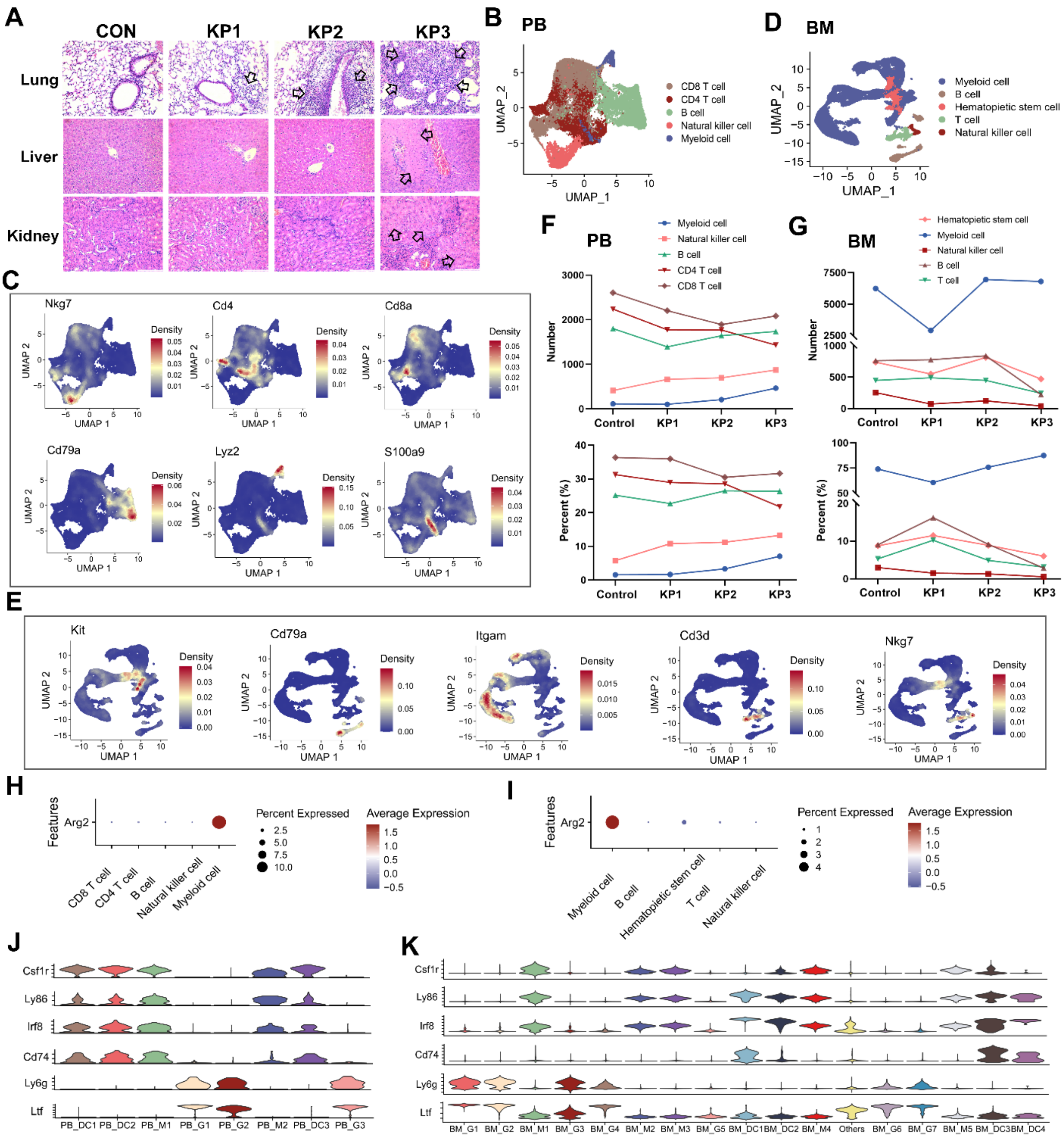


**Supplementary Figure 2. Supplementary data of sc-RNA seq analysis.** (A) Representative HE results of lung, liver, and kidney tissues from the PIS model at CON, KP1, KP2, and KP3 four stages. Black arrows indicated pathological areas. **(B)** After quality control, the two-dimensional UMAP distribution for 26,088 sequenced PBMCs was divided into five main types of immune cells. **(C)** Classical genes for cell-type identification in PBMC, *Nkg7* for natural killer cells, *Cd4* for CD4 T cells, *CD8a* for CD8 T cells, *Cd79a* for B cells, and *Lyz2* and *S100a9* for myeloid cells. **(D)** The two-dimensional UMAP distribution for 30,171 sequenced BM cells after quality control, which were divided into five main cell types. **(E)** Classical genes for cell-type identification in BM cells, while *Kit* for hematopoietic stem cells, *Cd79a* for B cells, *Cd3d* for T cells, *Nkg7* for natural killer cells, and *Itgam* for myeloid cells. **(F and G)** The number and proportion of main cell types in **F** PB and **G** BM with PIS progression.

**(H and I)** The dotplot exhibited the dominant *Arg2* expression in myeloid cells rather than other cell types in **H** PB and **I** BM samples. **(J and K)** The violin plot displayed the expression levels of classical genes in **J** PB- and **K** BM-derived myeloid clusters, while the *Irf8* and *Cd74* for dendritic clusters, the *Csf1r* and *Ly86* for monocyte clusters, and the *Ltf* as well as *Ly6g* for granulocyte clusters.

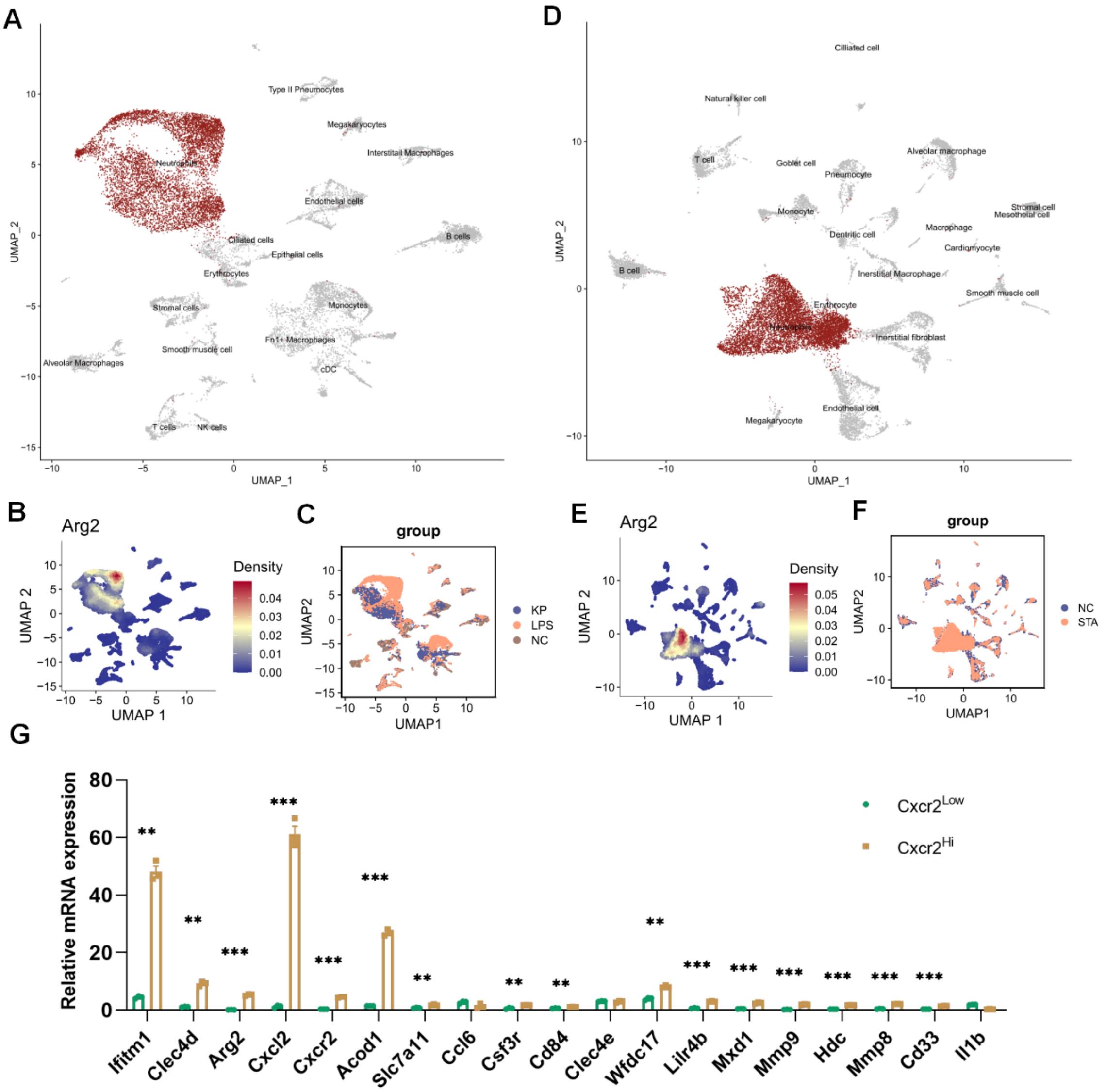

**Supplementary Figure 3.** ***Arg2*** **was enriched in granulocyte subsets in lung tissue from LPS- and bacteria-stimulated mice. (A-C)** The **A** two-dimensional UMAP distribution of lung samples from **B** *K.p* or LPS intratracheally inoculated and control (NC) mice in public sc-RNA dataset GSE190262, while the **C** *Arg2* transcript was primarily expressed in neutrophils. **(D-F)** The **D** two-dimensional UMAP distribution of lung samples from **E** staphylococcus aureus intratracheally inoculated and control (NC) mice in public sc-RNA dataset GSE215195, while the **F** *Arg2* transcript was primarily expressed in neutrophils. **(G)** RT-PCR assay showing the high expression of selected signature genes of ARG2 enriched MDSCs in the CXCR2$^{Hi}$ subset from KP mice. Statistical significances were calculated by unpaired t-test. Data were presented as mean ± SD. $**p < 0.01$, $***p < 0.001$.

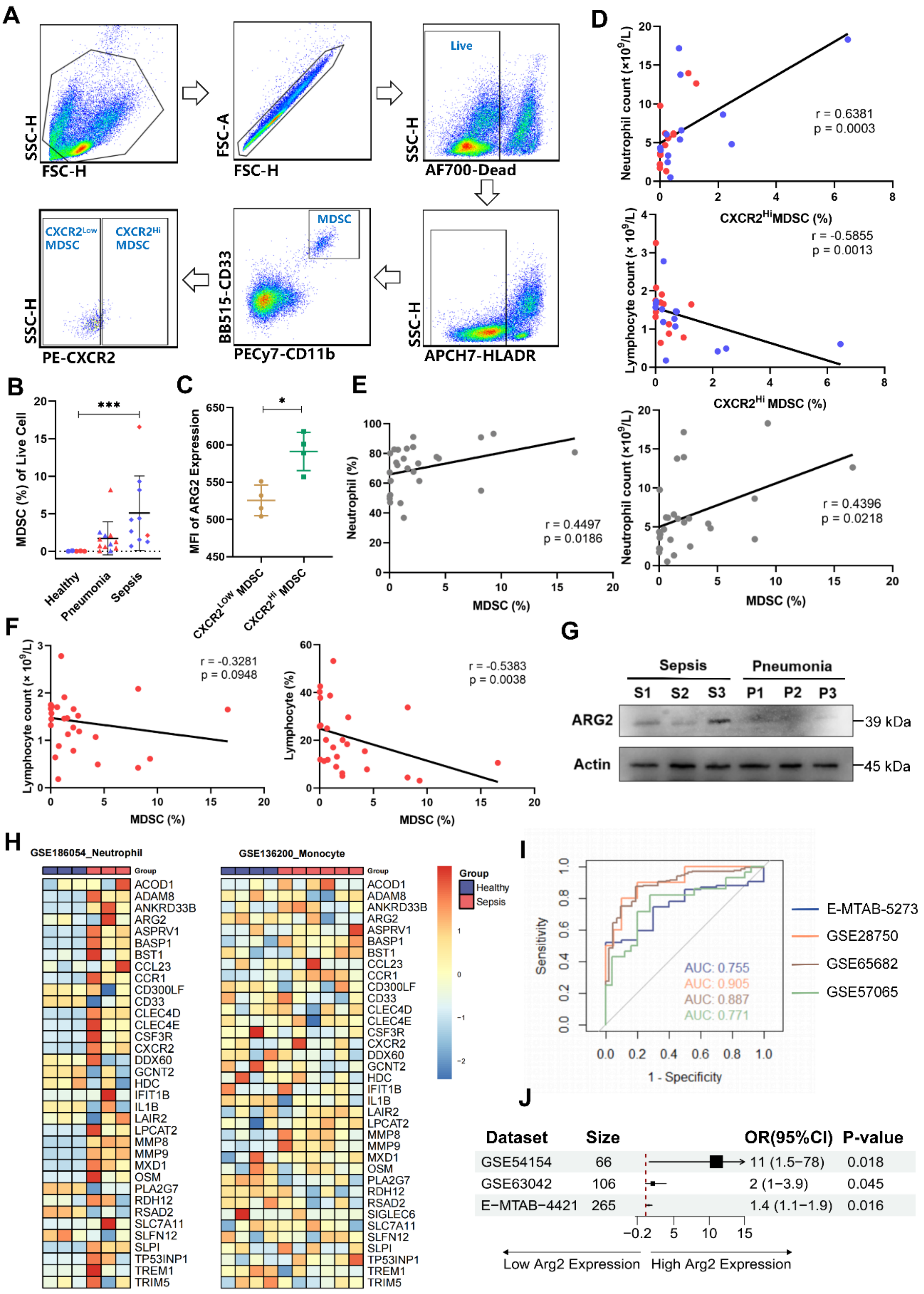


**Supplementary Figure 4. The ARG2 enriched CXCR2$^{Hi}$ MDSCs associated with low lymphocyte counts and poor prognosis in clinical scenarios and suppressed the proliferation of CD4+ T cells through its type II arginase role *in vitro*. (A)** Gating strategy of HLA-DR$^{Low}$CD11b$^{+}$CD33$^{+}$ MDSCs and HLA-DR$^{Low}$CD11b$^{+}$CD33$^{+}$CXCR2$^{Hi}$ MDSCs in white blood cells from clinical patients, as

detected by flow cytometry. **(B)** Statistics of proportions of total MDSCs between healthy volunteers (n = 5), pneumonia patients (n = 12), and septic patients (n = 10), as calculated by the Mann-Whitney test. Red and blue dots represented female and male individuals, respectively. **(C)** The flow cytometry detected the higher ARG2 expression in $CXCR2^{Hi}$ MDSCs than in the $CXCR2^{Low}$ subset. **(D)** A significant positive correlation between the percentage of $CXCR2^{Hi}$ MDSCs and neutrophil counts and a negative correlation between the percentage of $CXCR2^{Hi}$ MDSCs and lymphocyte counts. Red and blue dots represented female and male individuals, respectively. **(E)** Correlations between the percentage of MDSCs and neutrophil count and neutrophil proportion. **(F)** Correlations between the percentage of MDSCs and lymphocyte count and lymphocyte proportion. **(G)** Protein levels of ARG2 were higher in blood samples from the PIS than the pneumonia (n = 3 patients/group). **(H)** Signature genes of $CXCR2^{Hi}$ MDSC expressed highly in neutrophils (GSE186054) rather than in monocytes (GSE136200) from septic patients. **(I)** The receiver operator curves (ROC) for applying *Arg2* expression in sepsis diagnosis in four bulk transcriptome datasets, with all areas under curve (AUCs) more than 0.75. **(J)** The high *Arg2* expression were associated with 28-day death events in three datasets. Odds ratio (OR) was calculated by Logistic univariate regression. Data were presented as mean ± SD. ***$p < 0.001$.

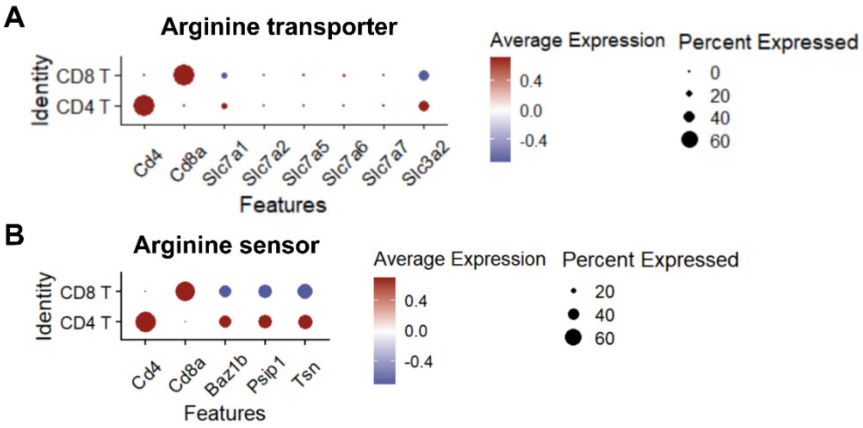


**Supplementary Figure 5. (A and B)** The dot plot showing differences in expression levels of **A** arginine transporters and **B** arginine sensors in splenic $CD4^+$ and $CD8^+$ T cells.

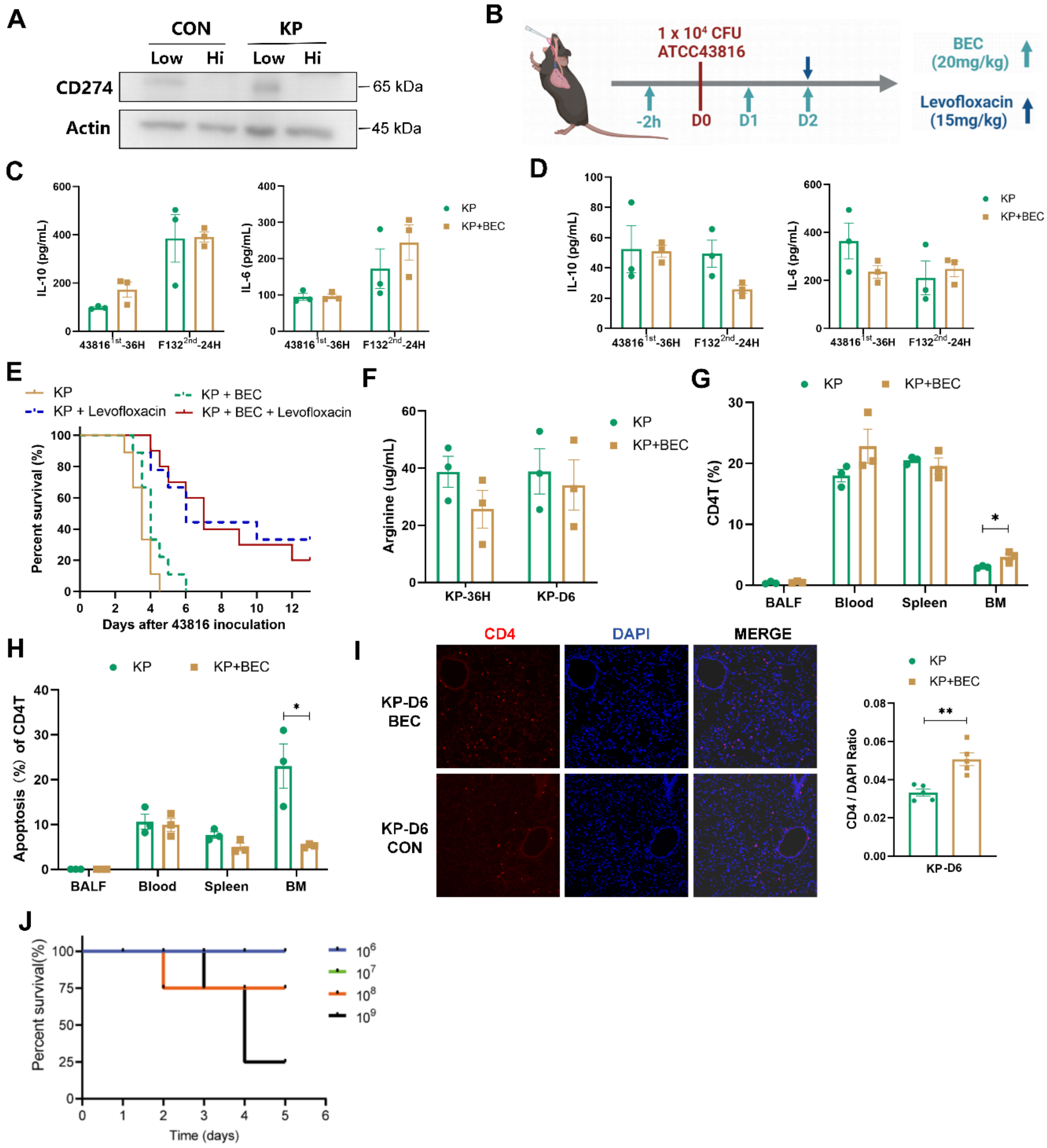


**Supplementary Figure 6. ARG2 inhibitor BEC did not impact inflammatory responses and outcomes in first infection. (A)** Protein expression level of immune checkpoint CD274 (PDL1) in CXCR2$^{Low}$ MDSC and CXCR2$^{Hi}$ MDSC by western blotting. **(B)** Schematic diagram of the treatment and sample collection procedures for PIS animal experiments, with infection of *K.p* stain #ATCC43816. **(C and D)** Concentrations of IL-10 and IL-6 in **C** BALF and **D** plasma samples at 36 hours after first infection and at 24 h after secondary infection. Patients. n = 3 biologically independent mice. **(E)** Kaplan-Meier survival curve of four mice groups (n = 8 mice/group) including KP, KP with BEC administration, and both with levofloxacin treatment or not. **(F)** The arginine concentration in plasma at 36 h and Day 6. n = 3 biologically independent mice. **(G and H)** Statistics on the **G** CD4$^{+}$ T cell percentage and its **H** apoptotic rates in BALF, BM, spleen, and blood at 36 h after *K.p* inoculation. Statistical significance is calculated by unpaired t-test. n = 3 biologically independent

mice. **(I)** Representative results (left) and statistics (right) of the immunofluorescence of lung tissue at Day 6 after *K.p* inoculation. Red indicated CD4, and blue indicated DAPI. **(J)** The survival rate of mice inoculating with F132 ($1\times10^6$, $1\times10^7$, $1\times10^8$ and $1\times10^9$ CFU) (n = 6 mice/group). Statistical significances were calculated by unpaired t-test. Data are presented as mean ± SD. * $p < 0.05$, **$p < 0.01$.

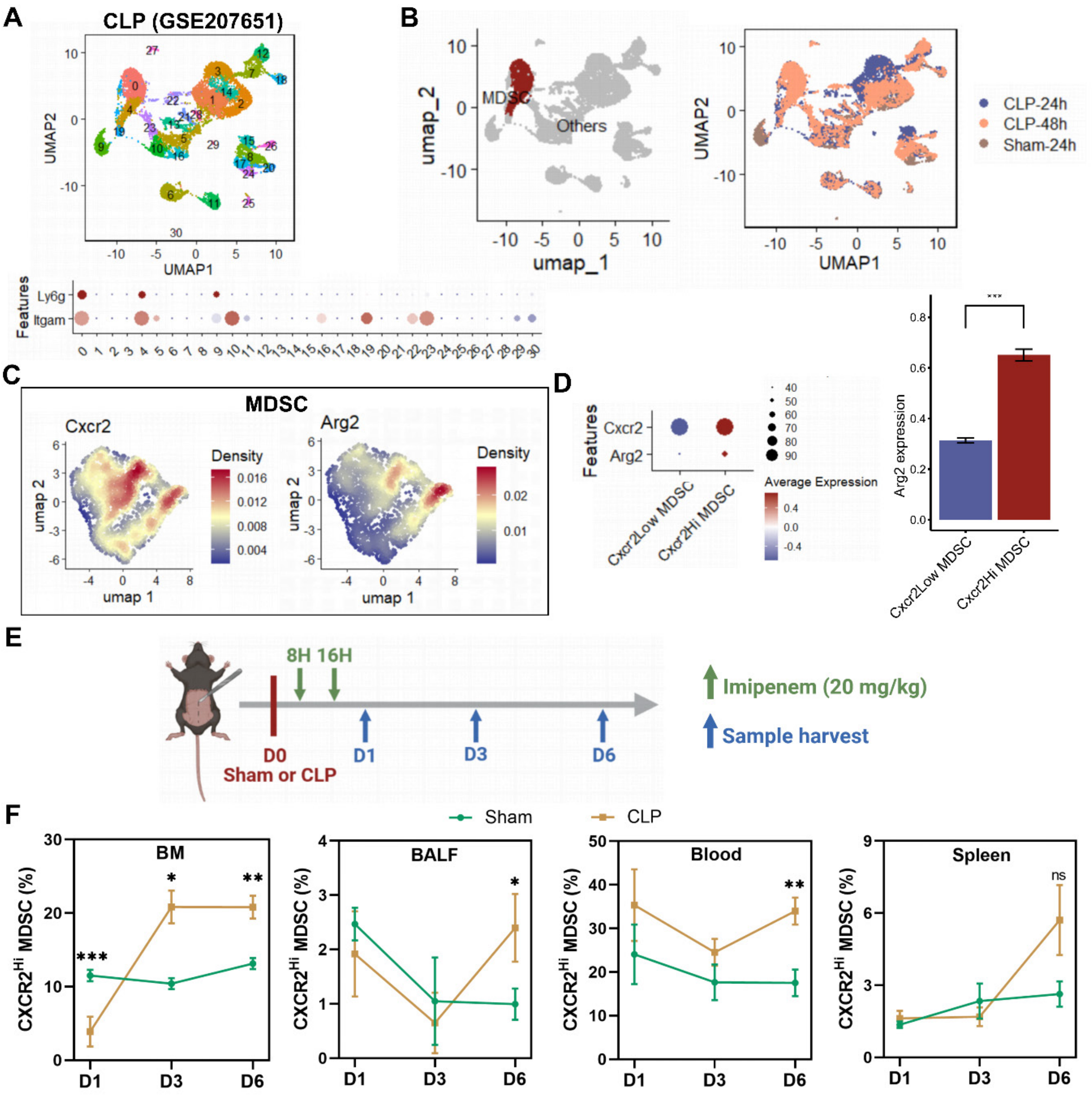


**Supplementary Figure 7. The ARG2-enriched CXCR2$^{Hi}$ MDSCs increased at a later stage in another septic model of cecum ligation and puncture (CLP). (A)** The two-dimensional UMAP distribution of lung samples from CLP mice in public sc-RNA dataset GSE207651, while the cluster 0 and cluster 4 highly expressed MDSCs' marker genes *Ly6g* and *Cd11b*. **(B)** The MDSCs (cluster 0 and cluster 4) increased in the CLP rather than the Sham. **(C)** The expression and distribution of *Cxcr2* and *Arg2* transcripts in MDSCs. **(D)** Similar to the PIS model, in the CLP model, the *Arg2* transcript was significantly enriched in the MDSC subset with high *Cxcr2* expression. The statistical significance was analyzed using the Wilcoxon test. **(E)** Schematic diagram of the antibiotic treatment and sample collection procedures for CLP animal experiments. **(F)** The dynamic detection of CXCR2$^{Hi}$ MDSCs in BM, BALF, blood, and spleen showed their accumulation at D6 after surgery. n = 3-4 biologically independent mice. Statistical significances were calculated by the unpaired t-test. *$p < 0.05$, **$p < 0.01$, ***$p < 0.001$.

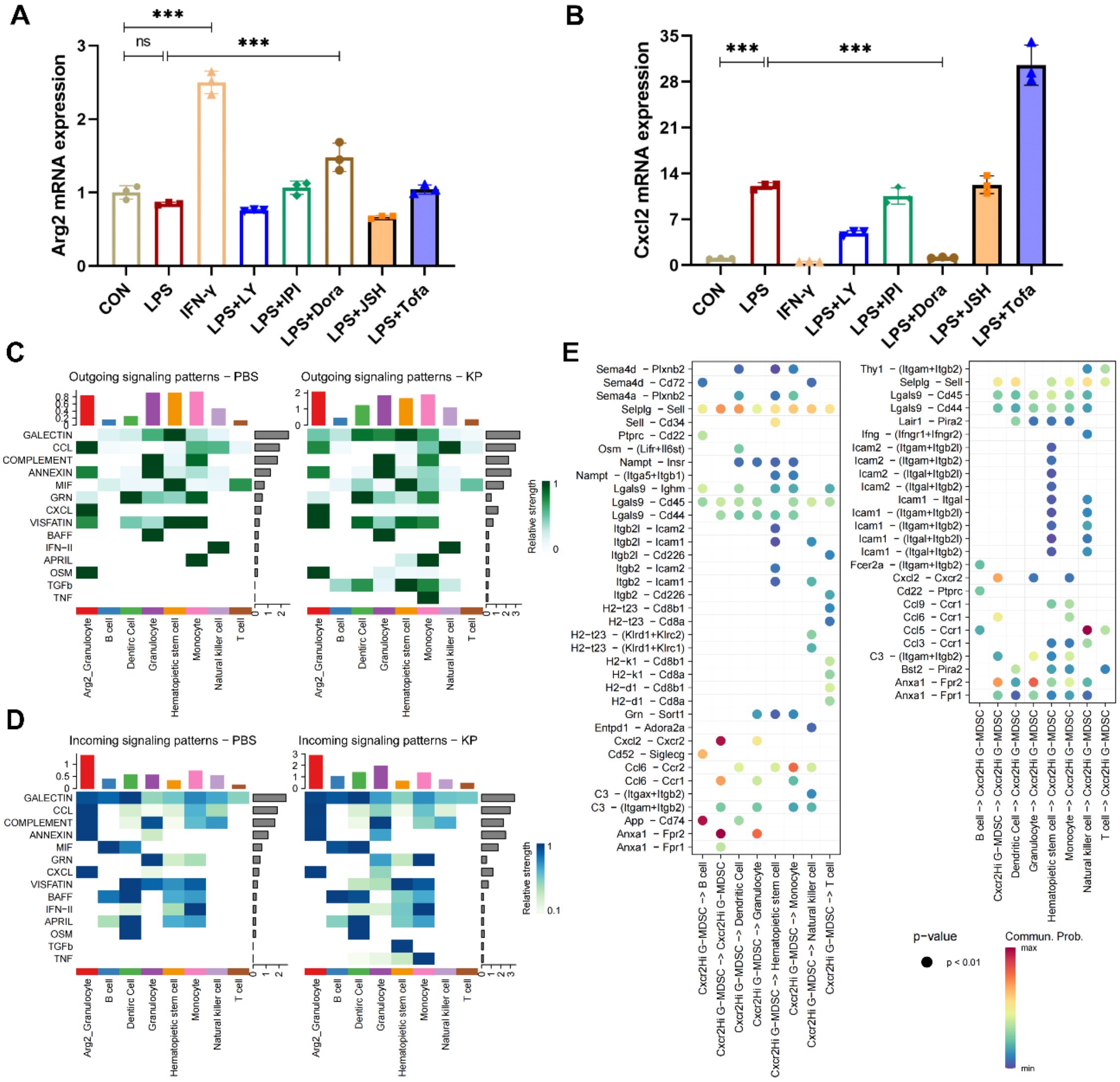


**Supplementary Figure 8. The cell-cell communication analysis predicted substantial Cxcl2-Cxcr2 crosstalk between ARG2-enriched CXCR2$^{Hi}$ MDSCs and itself. (A and B)** The mRNA expression levels of **A** *Arg2* and **B** *Cxcl2* in CXCR2$^{Hi}$ MDSCs after treatment with LPS, IFN-γ, and pathway inhibitors. LY: The PI3Kα, PI3Kδ, and PI3Kβ inhibitor LY294002. IPI: The PI3Kγ inhibitor IPI594. Dora: The p38-MAPK inhibitor Doramapimod. JSH: The NF-κB inhibitor JSH-23. Tofa: The JAK inhibitor Tofacitinib. Data were represented as mean ± SD.**(C and D)** The heatmap showed overall **C** incoming and **D** outgoing signaling of cell-cell communications in all cell types at CON (PBS) and KP stages. **(E)** The dot plot showed cell-cell communication probabilities between CXCR2$^{Hi}$ MDSCs as the sender (left) or target (right) and other cell types, including B cells, dendritic cells, granulocytes, hematopoietic stem cells, monocytes, natural killer cells, and T cells.

**Supplementary Tables**

**Supplementary Table 1. The assessment items and judging standards of pneumonia-induced sepsis (PIS) mice.**

| Assessment items | Manifestations of the pneumonia-induced sepsis |
|---|---|
| Temperature | < 32℃ |
| Behaviors | |
| Fur aspect | Piloerection, tarnished |
| Posture | Hunched and curled up |
| Breath state | Severely dyspnea with thoracic abdominal respiration |
| Activity | No relocation when stimulated† |
| Eye lids | Closed in usual, slightly open when stimulated* |

*: Stimulated state: Lid off the box and gently touched mice. Mice met all above criteria were identified as the PIS mouse.

**Supplementary Table 2. The body temperature in survived mice inoculation with PBS (control) and *K.p* ($2\times10^3$, $2\times10^4$, and $2\times10^5$ CFU).**

| | | Survived mice (n) | 1 | 2 | 3 | 4 | 5 | 6 | 7 | 8 | 9 | 10 |
|---|---|---|---|---|---|---|---|---|---|---|---|---|
| 0h | PBS | 10 | 37.2 | 37.4 | 37.9 | 37.4 | 37.6 | 37.9 | 37.3 | 37.9 | 38 | 37.6 |
| | $2\times10^3$ | 10 | 37.9 | 37 | 38.2 | 37.9 | 37.4 | 37.7 | 37.6 | 37.4 | 38 | 37.2 |
| | $2\times10^4$ | 10 | 36.9 | 38.2 | 37.0 | 37.1 | 37.9 | 37.3 | 37.2 | 38.1 | 37.8 | 37.5 |
| | $2\times10^5$ | 10 | 37.5 | 37.3 | 37.6 | 38 | 37.1 | 37.9 | 37.1 | 38 | 37.5 | 37.9 |
| 12h | PBS | 10 | 37.3 | 37.8 | 38 | 37.3 | 37.5 | 37.4 | 38.1 | 37.6 | 37.1 | 37.6 |
| | $2\times10^3$ | 10 | 37.1 | 37.7 | 38 | 36.7 | 38 | 37.8 | 37.6 | 37.2 | 37.2 | 36.9 |
| | $2\times10^4$ | 10 | 37 | 36.8 | 37.1 | 37.8 | 37.4 | 37 | 36.4 | 37.2 | 36.6 | 36.1 |
| | $2\times10^5$ | 10 | < 32 | < 32 | 35.2 | 36 | 35.4 | 35.6 | 35.2 | 34.8 | 34.9 | 35.1 |

| | | | | | | | | | | | | |
|---|---|---|---|---|---|---|---|---|---|---|---|---|
| 24h | PBS | 10 | 38.1 | 37.1 | 36.9 | 37.5 | 37.6 | 37.8 | 37.8 | 36.9 | 37.3 | 37.5 |
| | $2\times10^3$ | 10 | 37.2 | 38 | 36.4 | 37.2 | 37.3 | 37.3 | 37.6 | 37.2 | 36.9 | 37.1 |
| | $2\times10^4$ | 10 | 36.3 | 35.9 | 36.7 | 36.8 | 35.1 | 37 | 35.9 | 36.3 | 36 | 35.8 |
| | $2\times10^5$ | 10 | < 32 | < 32 | < 32 | 33.3 | 34.2 | 34.9 | 35.8 | 32.5 | 33.4 | 33.1 |
| 36h | PBS | 10 | 36.6 | 36.9 | 38.2 | 38 | 37.8 | 36.8 | 37.6 | 37.1 | 37.9 | 38 |
| | $2\times10^3$ | 10 | 37.3 | 36.9 | 37.1 | 37.3 | 36.5 | 37 | 35.9 | 36.4 | 37.5 | 36.9 |
| | $2\times10^4$ | 10 | < 32 | < 32 | 34 | 34.7 | 33.6 | 35.5 | 35.8 | 36 | 35.2 | 33.4 |
| | $2\times10^5$ | 9 | < 32 | < 32 | < 32 | < 32 | < 32 | < 32 | < 32 | 33.5 | 32.9 | |
| 48h | PBS | 10 | 37.6 | 38.0 | 37.2 | 37.0 | 37.6 | 37.9 | 37.7 | 37.3 | 37.7 | 38.1 |
| | $2\times10^3$ | 10 | 37 | 36.7 | 37.1 | 36.4 | 36.5 | 37.8 | 36.6 | 35.4 | 36.5 | 34.9 |
| | $2\times10^4$ | 9 | < 32 | < 32 | 35.8 | 34 | 35.2 | 35.7 | 33.4 | 36.2 | 32.3 | |
| | $2\times10^5$ | 6 | < 32 | < 32 | < 32 | < 32 | < 32 | 32.4 | | | | |
| 60h | PBS | 10 | 37.9 | 37.4 | 37 | 37.5 | 36.8 | 37.9 | 37.3 | 38.1 | 37.9 | 37.4 |
| | $2\times10^3$ | 10 | < 32 | < 32 | 35.6 | 33.5 | 37 | 36.4 | 34.7 | 37.2 | 33.9 | 32.9 |
| | $2\times10^4$ | 7 | < 32 | < 32 | 33 | 34.6 | 33.8 | 35.1 | 34.2 | | | |
| | $2\times10^5$ | 4 | < 32 | < 32 | < 32 | < 32 | | | | | | |
| 72h | PBS | 10 | 37.3 | 37 | 37.1 | 37.4 | 38.1 | 37.6 | 37.6 | 36.9 | 37.4 | 37.2 |
| | $2\times10^3$ | 10 | < 32 | < 32 | < 32 | < 32 | < 32 | 35.3 | 36.9 | 35.8 | 36.5 | 35.7 |
| | $2\times10^4$ | 4 | < 32 | < 32 | 32.2 | 32.6 | | | | | | |
| | $2\times10^5$ | 0 | | | | | | | | | | |

**Supplementary Table 3. Characteristics of public transcriptomic datasets included in this study.**

| Dataset | Download platform | Specie | Experiment type | Sample |
|---|---|---|---|---|
| GSE190262 | GEO | mouse | scRNA-seq | Lung (*K.p* infected and LPS administrated) |
| GSE215195 | GEO | mouse | scRNA-seq | Lung (STA infected) |
| GSE207651 | GEO | mouse | scRNA-seq | Lung (Sham and CLP) |
| GSE222784 | GEO | mouse | scRNA-seq | Spleen (healthy) |
| GSE186054 | GEO | human | RNA-Seq | Neutrophil (sepsis) |
| GSE136200 | GEO | human | RNA-Seq | Monocyte (sepsis) |
| E-MTAB-5273 | ArrayExpress | human | Array | Whole-blood leukocyte (sepsis) |
| GSE28750 | GEO | human | Array | Whole-blood leukocyte (sepsis) |
| GSE65682 | GEO | human | Array | Whole-blood leukocyte (sepsis) |
| GSE57065 | GEO | human | Array | Whole-blood leukocyte (sepsis) |
| GSE54154 | GEO | human | Array | Whole-blood leukocyte (sepsis) |
| GSE63042 | GEO | human | RNA-Seq | Whole-blood leukocyte (sepsis) |
| E-MTAB-4421 | ArrayExpress | human | Array | Whole-blood leukocyte (sepsis) |

**Supplementary Table 4. Gene lists for functional scoring of ARG2 enriched CXCR2$^{Hi}$ MDSCs.**

| MDSC | Specific_granules | NADPH_oxidase | Azurophil_granulesb |
|---|---|---|---|
| Wfdc17 | Lyz2 | Cybb | Hexa |
| Ifitm1 | Lcn2 | Cyba | Prss57 |
| Il1b | Cyba | Rac2 | Prtn3 |
| BC100530 | Cybb | Rac1 | Ctsg |
| Srgn | Ncf1 | Ncf2 | Elane |
| Gm5483 | Ncf4 | Ncf1 | Mpo |
| Stfa2l1 | Ltf | Ncf4 | Ctsc |
| Stfa2 | Camp | | |
| Prok2 | | | |
| Ifitm2 | | | |
| Junb | | | |
| Dusp1 | | | |
| Socs3 | | | |
| Btg1 | | | |
| 1600014C10Rik | | | |
| Selplg | | | |
| Asprv1 | | | |
| Igfbp6 | | | |
| Gm5150 | | | |
| Pla2g7 | | | |

| |
|---|
| Csf3r |
| Cxcr2 |
| Tpd52 |
| Tspo |
| Cyp4f18 |
| Grina |
| Fabp5 |
| Clec4d |
| Steap4 |
| Map1lc3b |
| Ccr1 |
| Lrg1 |
| Clec4e |
| Ctsd |
| Cd84 |
| Gcnt2 |
| Arg2 |
| BC117090 |
| Npl |
| Fgl2 |
| Rnf149 |
| Sephs2 |
| S100a6 |
| Lmnb1 |
| Amica1 |
| Eif4ebp1 |
| Msrb1 |
| Ubb |
| C5ar1 |
| Ypel3 |
| Fcgr3 |
| Gsr |
| Taldo1 |
| Atp6v1g1 |
| S100a11 |
| Hp |
| Alox5ap |
| Litaf |
| Txn1 |
| Upp1 |
| AB124611 |
| Gpcpd1 |
| Snap23 |
| Il4ra |

| |
|---|
| Hist1h2bc |
| Retnlg |
| Myd88 |
| Adipor1 |
| Stk17b |
| Zyx |
| Rgs3 |
| Il1f9 |
| Hdc |
| Atg3 |
| Gda |
| Slc40a1 |
| Tarm1 |
| Cdk2ap2 |
| Stfa3 |
| Glipr2 |
| Tacstd2 |
| Picalm |
| Mtus1 |
| Fbxl5 |
| Slfn1 |
| Rnd1 |
| Ier2 |
| Mxd1 |
| Siglece |
| Cdkn2d |
| Cd33 |
| Lilr4b |
| Cd14 |
| Sell |
| Ppt1 |
| Skap2 |
| Sfxn5 |
| 2810474O19Rik |
| Ikbkap |
| Hbb-bs |
| Atp11b |
| Cxcl2 |
| Osm |
| Ier3 |
| Hba-a1 |

**Supplementary Table 5. Signature genes of ARG2 enriched CXCR2$^{Hi}$ MDSCs, as generated by COSG and Findmarker.**

| Gene | COSG score | Findmarker avg_log2FC | Findmarker p_val_adj |
|---|---|---|---|
| Acod1 | 0.482509878 | 0.883351576 | 0 |
| Adam19 | 0.311860106 | 0.701278309 | 0 |
| Adam8 | 0.26961558 | 0.775699974 | 0 |
| Ankrd33b | 0.433460111 | 0.641799542 | 2.57E-214 |
| Asprv1 | 0.254473467 | 0.573986622 | 7.66E-140 |
| Basp1 | 0.2201211 | 0.526687165 | 2.61E-284 |
| Bst1 | 0.256930602 | 0.793884771 | 0 |
| Ccl6 | 0.214639173 | 0.945517684 | 0 |
| Ccr1 | 0.228939268 | 0.808657155 | 0 |
| Cd300ld | 0.392502449 | 0.766766799 | 0 |
| Cd33 | 0.242934183 | 0.91289871 | 0 |
| Clec4d | 0.667115114 | 1.190757812 | 0 |
| Clec4e | 0.270275374 | 0.83313437 | 0 |
| Csf3r | 0.29936599 | 0.921790624 | 0 |
| Cxcl2 | 0.511397525 | 1.396866626 | 0 |
| Cxcr2 | 0.345942951 | 1.117188641 | 0 |
| Ddx60 | 0.310148155 | 0.766520132 | 0 |
| Dusp1 | 0.190028443 | 0.792135082 | 0 |
| Gcnt2 | 0.249138911 | 0.612971562 | 3.22E-246 |
| Hdc | 0.27458751 | 1.007490567 | 0 |
| Ifit1 | 0.2530592 | 0.543611083 | 7.69E-128 |
| Ifitm1 | 0.437906303 | 1.036703285 | 0 |
| Il1b | 0.424069919 | 1.02966685 | 0 |
| Il1rn | 0.200209506 | 0.486095374 | 1.78E-268 |
| Lilr4b | 0.259126457 | 0.922138263 | 0 |
| Lpcat2 | 0.196030948 | 0.57688202 | 3.35E-219 |
| Mmp8 | 0.333321141 | 1.201330099 | 0 |
| Mmp9 | 0.275491208 | 1.177669746 | 0 |
| Mxd1 | 0.201604232 | 0.954805129 | 0 |
| Oasl2 | 0.305812379 | 0.662344916 | 2.97E-234 |
| Osm | 0.283743976 | 0.779442319 | 0 |
| Pla2g7 | 0.39287893 | 0.560911256 | 1.81E-172 |
| Rdh12 | 0.230947726 | 0.636047995 | 8.08E-259 |
| Retnlg | 0.455043273 | 1.630525772 | 0 |
| Rsad2 | 0.294173352 | 0.807244596 | 0 |
| Slc7a11 | 0.414003548 | 1.145293412 | 0 |
| Slfn1 | 0.205490753 | 0.687369064 | 0 |
| Slpi | 0.198002753 | 0.706446333 | 0 |
| Timp2 | 0.228287072 | 0.502645359 | 1.28E-118 |

| | | | |
|---|---|---|---|
| Trem1 | 0.2476877 | 0.735769243 | 0 |
| Trim30b | 0.384767346 | 0.818017842 | 0 |
| Trp53inp1 | 0.254166001 | 0.648812225 | 3.57E-278 |
| Wfdc17 | 0.316257379 | 0.794235914 | 1.84E-270 |

**Supplementary Table 6. Demographics of clinical patients in this study.**

| Characteristics | Healthy (n = 5) | Pneumonia (n =12) | Sepsis (n = 10) | p value |
|---|---|---|---|---|
| **Age, median (IQR)** | 54.00 (49.00, 60.00) | 45.50 (28.50, 61.50) | 68.50 (55.00, 72.00) | 0.102 |
| **Sex, n (%)** | | | | 0.091 |
| Male | 2 (40.0%) | 4 (33.3%) | 8 (80.0%) | |
| **Medical history** | | | | |
| Hypertension, n (%) | 2 (40.0%) | 1 (8.33%) | 3 (30.0%) | 0.276 |
| Diabetes, n (%) | 1 (20.0%) | 0 (0%) | 3 (30.0%) | 0.113 |
| Chronic pulmonary disease, n (%) | 4 (80.0%) | 0 (0%) | 6 (60.0%) | <0.001 |
| Chronic cardiac disease, n (%) | 1 (20.0%) | 0 (0%) | 6 (60.0%) | 0.003 |
| Chronic liver disease, n (%) | 0 (0%) | 1 (8.33%) | 5 (50.0%) | 0.041 |
| Chronic kidney disease, n (%) | 0 (0%) | 1 (8.33%) | 0 (0%) | >0.999 |
| Solid malignant tumor, n (%) | 1 (20.0%) | 0 (0%) | 3 (30.0%) | 0.113 |
| **Laboratory findings** | | | | |
| Lymphocyte count (× $10^9$ /L), median (IQR) | 1.72 (1.67, 1.75) | 1.55 (1.24, 1.75) | 0.93 (0.52, 1.39) | 0.007 |
| Neutrophil count (× $10^9$ /L), median (IQR) | 3.60 (2.22, 4.04) | 5.81 (3.39, 6.18) | 7.61 (4.95, 13.50) | 0.078 |
| C-reactive protein (mg/L), median (IQR) | 2.72 (0.50, 3.17) | 4.91 (2.80, 24.09) | 27.74 (9.65, 125.58) | 0.068 |
| Procalcitonin (mg/L), median (IQR) | NA | NA | 14.31 (2.50, 98.50) | - |
| **Outcomes** | | | | 0.025 |
| Survival, n (%) | 5 (100%) | 12 (100%) | 6 (60%) | |

**Supplementary Table 7. Mouse-specie primers used in this study.**

| Gene | Forward | Reverse |
|---|---|---|
| Arg1 | CTCCAAGCCAAAGTCCTTAGAG | AGGAGCTGTCATTAGGGACATC |
| Arg2 | AGGAGTGGAATATGGTCCAGC | AGGGATCATCTTGTGGGACATT |
| CD84 | ATATAGCTGGAGTCCCTTTGGAG | AAAGAGCACGGCCAATCCTC |
| Clec4d | CCTGCTGTCCTGTTAGCTGG | CTGTTCTGCTTCGGTGTTGAT |
| Wfdc17 | TTTGATCACTGTGGGGATG | ACACTTTCTGGTGAAGGCTTG |
| Clec4e | AGTGCTCTCCTGGACGATAG | CCTGATGCCTCACTGTAGCAG |
| Cxcr2 | ATGCCCTCTATTCTGCCAGAT | GTGCTCCGGTTGTATAAGATGAC |
| Ifitm1 | GACAGCCACCACAATCAACAT | CCCAGGCAGCAGAAGTTCAT |
| Csf3r | CTGATCTTCTTGCTACTCCCCA | GGTGTAGTTCAAGTGAGGCAG |
| Slc7a11 | AAGTCTAATGGGGTTGCCCT | TGATAGCCATGGAGATGCAG |
| Mmp8 | TCAACCAGGCCAAGGTATTG | ATGAGCAGCCACGAGAAATAG |
| Mmp9 | TTGGTTTCTGCCCTAGTGAGAGA | AAAGATGAACGGGAACACACAGG |
| Cxcl2 | AAGTTTGCCTTGACCCTGAA | AGGCACATCAGGTACGATCC |
| Ccl6 | ATGATGAGACATTCCAAGACTGC | TCAAGCAATGGCACTGTTCCCAGA |
| Hdc | GCGACCCTTCCTTCGAAATT | CCTTTAACACACTTTCTGTGAGACAAT |
| Cd33 | CCGCTGTTCTTGCTGTGTG | AAGTGAGCTTAATGGAGGGGTA |
| Lilr4b | AGTGTCGTCACAAAAATAAGGCT | CCTGGGCGTACACAATTCCC |
| Mxd1 | AGATGCCTTCAAACGGAGGAA | CAAGCTCAGAGTGGTGTGTCG |
| Il1b | ACAGCAATGATGAGGATCTGC | CTCTAGGTGGGTCTTGGGAAC |
